# Understanding the “how” and “why”: A mixed methods process evaluation for the PRO-HIIT intervention

**DOI:** 10.1371/journal.pone.0352772

**Published:** 2026-06-30

**Authors:** Yong Liu, Alan R. Barker, Minghui Li, Anna-Lynne R. Adlam, Stephanie L. Duncombe, Andrew O. Agbaje, Yaodong Gu, Huiyu Zhou, Craig A. Williams

**Affiliations:** 1 Sports and Arts Department, Zhejiang Wanli University, Ningbo, China; 2 Children’s Health and Exercise Research Centre, Public Health and Sports Sciences, Faculty of Health and Life Sciences, University of Exeter, Exeter, United Kingdom; 3 Faculty of Sports Science, Ningbo University, Ningbo, China; 4 Psychology, Faculty of Health and Life Sciences, University of Exeter, Exeter, United Kingdom; 5 School of Public Health, The University of Queensland, Herston, QLD, Australia; 6 Institute of Public Health and Clinical Nutrition, School of Medicine, University of Eastern Finland, Kuopio Campus, Kuopio, Finland; Erzurum Technical University: Erzurum Teknik Universitesi, TÜRKIYE

## Abstract

**Introduction:**

Process evaluation completes outcome evaluation by explaining “how” and “why” an intervention is (in)effective. The aim of this study was to conduct a mixed methods process evaluation for the PRO-HIIT intervention.

**Methods:**

The PRO-HIIT intervention replaced the traditional warm-up period with 6–8 minutes of high-intensity interval training in the physical education and activity lessons, aiming to promote physical fitness, psychological parameters and academic performances among Chinese adolescents. The process evaluation was guided by the Medical Research Council guidance for the evaluation of complex interventions. Three process evaluation domains, including twelve process evaluation measures, were assessed using both quantitative and qualitative methods. Key means for process evaluation included were training logbook recording, intensity monitoring, and semi structured focus groups.

**Results:**

The PRO-HIIT intervention showed high level of retention rate and dose received. The dose delivered was slightly less than anticipated, with an average 26.5 sessions delivered over twelve intervention weeks. The average heart rate was 146 beats per minute, corresponding to 71% maximum heart rate, with the mean heart rate peak was 175 beats per minute (85% maximum heart rate). The average sessional rating of perceived exertion was 5, ranging from 3 to 8. Overall, participants and physical education teachers showed positive response towards the PRO-HIIT intervention. Session duration and work-to-rest ratio were adapted to balance the intervention satisfaction and effectiveness. Barriers to intervention delivery included competing priorities, severe weather, lack of sleep, repetition of exercises, and rating of perceived exertion administration, whereas facilitators included space efficiency, imparting knowledge, peer coaches, music, and physical education teachers perform the session with participants. No intervention-related injury occurred.

**Conclusions:**

This process evaluation provided a lens through which to facilitate the interpretation of the effectiveness of the PRO-HIIT intervention. The results provide valuable insights into how a school-based HIIT intervention can be implemented and refined.

## Introduction

Process evaluations, aimed at providing a more detailed understanding to inform policy and practice, are an essential component of designing and testing complex interventions (e.g., school-based interventions) [1]. While a randomised controlled trial (RCT) determines the effectiveness of an intervention at a specific time and context, a process evaluation facilitates the interpretation of findings and an understanding of how the intervention could be applied in other contexts. Incorporating a process evaluation in parallel with a RCT enables evaluators to minimise biases in estimating effects, as well as providing policymakers, practitioners, and/or systematic reviewers with insights into the effectiveness of an intervention [2]. However, process evaluations are often undervalued and underutilised compared to outcome evaluations in the literature of complex interventions.

School-based high-intensity interval training (HIIT) interventions are a type of complex intervention that have garnered significant interest in recent years. HIIT is defined as alternating brief bouts of high-intensity exercises (e.g., 80% maximum heart rate [HR_max_]) with periods of rest or active recovery [3]. HIIT has been adopted as a vehicle to deliver physical activity to children and adolescents due to several appealing features, such as time-efficiency [3,4], vigorous intensity [5,6], and mimicking the habitual activity patterns of young people [7]. Schools provide an ideal setting for accommodating HIIT interventions, given their abundant resources, such as staff, space and facilities, flexible scheduling options (e.g., breaks and classes), and broad reach of children and adolescents [8]. Meanwhile, schools are also complex institutions for implementing PA interventions, with challenges such as addressing the needs of different stakeholders (e.g., governors, teachers, parents and students) as well as navigating competing interests [8,9]. Indeed, the effectiveness of school-based interventions in increasing moderate to vigorous physical activity (MVPA) has been so far, limited [8–10].

In these circumstances, conducting a process evaluation alongside outcome assessments can provide valuable insights into aspects of implementation, mechanisms of impact, and the contextual factors that shape the (in)effectiveness of an intervention [2]. However, most school-based HIIT interventions have focused solely on outcome evaluation, with process evaluation largely overlooked [11]. A recent review of school-based HIIT interventions found that, out of 45 included interventions, only five explicitly included process evaluation as either a section or a separate piece of the outcome paper, and just one adopted a process evaluation framework [11]. Given that the reporting of process evaluation is likely to contribute on the intervention effectiveness [12,13], there is a clear need for greater emphasis on process evaluation in future interventions, which should be guided by an existing framework for a comprehensive evaluation [11].

The PRO-HIIT intervention is a two-arm cluster RCT conducted in a secondary school, aiming to promote physical fitness, executive function, well-being and academic performance among Chinese adolescents [14]. A brief HIIT session (6–8-minute) was incorporated into participants’ daily curriculum time for PA (e.g., physical education (PE) lessons) for 12 weeks. While effect sizes quantify the effectiveness of interventions, they offer limited insights into the underlying “how” and “why” – that is, how the implementation is carried out and why the intervention produced these outcomes (i.e., causal mechanisms and contextual factors) [2]. Therefore, the aim of the current study was to comprehensively evaluate the implementation process of the PRO-HIIT programme, with the guidance of the process evaluation of complex interventions using the Medical Research Council (MRC) guidance [1].

## Materials and Methods

### The PRO-HIIT intervention

The PRO-HIIT intervention has been described in detail in a published protocol paper [14], and registered on ClinicalTrials.gov Protocol Registration and Results System (NCT06374732) on 18/04/2024. The intervention is a multi-centre collaborative work conducted by University of Exeter and Ningbo University. Ethics approval was granted by the Ningbo University Ethics Committee (TY2024002). All participants and their parents or legal guardians have provided written assent and consent forms from 1^st^ to 8^th^ March 2024. All procedures were conducted in accordance with the ethical standards of these committees and with the Declaration of Helsinki. Three hundred and sixty-nine Year 7 students (aged 12–13 years) from eight different classes were included in the intervention, all of whom provided written assent and parental consent. The intervention adopted a two-arm cluster-RCT design, with an intervention group and a control group. Outcome variables were taken at three time points: pre-intervention (T1), immediately post-intervention (T2), and 2-month follow-up (T3). The intervention delivery was guided by the Supportive, Active, Autonomous, Fair, and Enjoyable (SAAFE) principles [15], with the details for the definition and how the principles applied to the PRO-HIIT intervention displayed in the protocol paper [14], as well as outlined in Table 1.

**Table 1:** Definition and application of SAAFE principles in the *PRO-HIIT* intervention.

Principles	Definition	Apply to *PRO-HIIT*	Result	Compliance
Supportive	Intervention is designed to facilitate a supportive environment	Encourage praise of students’ effort and improvement during HIIT sessions and outcome evaluation process.	These principles were included as the content of the training workshop for students, PE teachers and research assistants as relevant. However, the authors are unsure about the application of these principles as no on-site observation was conducted.	
		Encourage mutual support when performing HIIT and outcome assessments.		Unknown
		Demonstrate empathy toward students when they feel frustrated or challenged.		
Active	Sessions are highly active	Sessions are designed without any instruction time.	HIIT sessions were performed directly as led by peer coaches.	High
		Exercises are performed at high intensity.	Mean HR = 146 bpm; Mean HR_max_ = 175 bpm; Mean RPE = 5.	High
Autonomous	Sessions involve elements of choice	Right to play any music they like.	Partially complied by listening to students’ suggestions, while teachers made the decision.	Medium
		Right to choose exercises from an exercise pool (final 3 weeks).	No, teachers did not do so to facilitate the intervention delivery.	Low
		Perform the missed HIIT sessions themselves during breaks.	This was acknowledged during students’ training workshop. However, no students performed the missed sessions during the breaks.	Low
		Minimize controlling language.	Trained over the workshop, the application was unknown.	Unknown
Fair	Intervention provides all students with opportunities to experience success	Encourage self-comparison rather than peer-comparison.	Trained over the workshop, the application was unknown.	Unknown
		Provide personalised care for individuals with special needs (e.g., participants with lower fitness levels).	Students with lower fitness level or overweight/obese were allowed to finish outcome assessment at the end and the results were not exposed to other students. Teachers were instructed to individualise exercise intensity if necessary.	Medium
Enjoyable	Intervention is designed to be enjoyable and engaging for all students	Provide different HIIT workouts.	Multiple HIIT workouts were provided. However, more workouts are expected.	Medium
		Provide challenging HIIT sessions.	The session length increased from 6 to 8 minutes, but not 10 minutes.	Medium
		Play music while exercising.	Multiple different songs were used over the intervention period.	High
		Peer coaches to lead the HIIT sessions.	Each class had at least 2 peer coaches to lead the HIIT sessions.	High

HIIT, high-intensity interval training; PE, physical education; HR, heart rate; bpm, beats per minute; HR_max_, maximum heart rate; RPE, rating of perceived exertion.

A 6-to-10-minute HIIT session was embedded at the beginning of the PE (3 times/week) or PA (complementary lessons for PA on days when PE lessons were not scheduled, 2 times/week) lessons for the HIIT group, while the control group continued their traditional warm-up. This warm-up phase generally included 5–6 minutes of light intensity running followed by static stretches, totalling approximately 10 minutes. The intervention was planned to be delivered five times per week for twelve weeks, from March to June 2024.

The HIIT sessions consisted of body-weight resistance exercises selected based on previous research [16,17]. The work-to-rest ratio was designed to progressively increase within each session, and the session length was gradually extended from 6 to 10 minutes over the 12 weeks. A detailed plan of the intervention is provided in S1 File. The HIIT sessions were delivered by PE teachers with the assistance of two peer coaches in each intervention class. The peer coaches were trained to demonstrate and lead the HIIT sessions in front of the class, as well as help with other tasks, such as taking attendance, which varied at the discretion of the PE teachers. More details regarding the training workshops can be found in our protocol paper [14]. The researcher maintained regular contact with the PE teachers via online communication on a weekly basis and visited the intervention school once per month to provide ongoing support and guidance. To boost participant adherence and engagement, a prize draw offering university souvenirs was held upon completion of the intervention.

### Process evaluation measures

To improve the understanding of the intervention’s effectiveness and support its dissemination, a process evaluation was incorporated into the planning, design, implementation, and reporting stages of the PRO-HIIT intervention, as guided by the MRC guidance [1]. The MRC guidance was developed by a group of researchers with expertise in complex interventions through a series of workshops, conferences, and seminars [18]. Based on the MRC guidance and a recent review of school-based HIIT interventions [11], three process evaluation domains comprising twelve specific measures were identified to adapt the MRC guidance into the context of the PRO-HIIT intervention. The definition of the process evaluation domains and measures, and how these measures were evaluated are described in detail in the protocol paper [14]. A brief description and how these measures were evaluated is provided below.

#### Implementation.

*Reach* was defined as the target audience approached, which was quantified as the number of schools, PE teachers and participants who were contacted.

*Recruitment and retention* were documented as the number of participants who were included and completed the intervention, respectively.

*Dose delivered* was reported as how many HIIT sessions (and minutes per session) were delivered by the teachers and was recorded in a teacher’s training logbook. Of note, a minimum of 30 sessions was expected to be delivered over the 12-week period, taking into account the Chinese context (e.g., holidays and weather) as well as informed by a previous intervention [19].

*Adaptation* was defined as alterations made to the intervention to achieve better contextual fit, which was documented as changes from the protocol to facilitate the PRO-HIIT intervention delivery. This was reported in the training logbook as well as over a focus group with teachers.

*Fidelity* was defined as intervention delivered as intended, which was evaluated as: 1) the extent to which the intervention complied with the SAAFE principles; 2) session intensity. To minimise burden, session intensity was monitored in only two randomly selected classes and for once per week during PE lessons. PA lessons were not monitored due to a lack of personnel. In addition, one of the selected classes was monitored through HR with the Polar Verity Sense, while the other class using the OMNI rating of perceived exertion (RPE) scale. Both tools are validated for use in the context of HIIT in adolescents [20–22]. Due to the limited availability of HR monitors, HR data were collected from only ten participants in each session. The mean HR and peak HR achieved were reported, whereas RPE was recorded as the sessional RPE rating. The HIIT intensity threshold was set at HR ≥ 80% HR_max_ (with individual HR_max_ calculated using 220-age) or RPE ≥ 5 [20].

#### Mechanisms of impact.

*Dose received* was defined as the number and the quality (engagement) of the HIIT sessions performed by participants. This was assessed using attendance and PE teacher ratings of session quality on a 0–10 scale, with both measures recorded in the training logbook. In addition, the session quality was also assessed qualitatively through student and teacher focus groups.

*Response* was the students and teachers’ reaction and attitude towards the PRO-HIIT intervention. It was assessed qualitatively via student and teacher’s focus groups as well as quantitatively on a 2-item 5-point Likert scale, which has been used in previous school-based HIIT interventions for assessing satisfaction [23,24]. The scale started with the prompt: “I enjoyed/liked the HIIT workouts” and “I will continue to perform/use the HIIT workouts”, with the score ranged between 1 = strongly disagree and 5 = strongly agree. Both teachers and students answered the scale at T2.

*Unintended consequence* was defined as any unexpected issues that happened over the intervention period, which may shape the trajectory of the intervention outcome. This was reported as HIIT-related injuries (via training logbook), as well as any other unintended issues that impacted the intervention.

#### Context.

*Barriers and facilitators* were the factors that undermine or facilitate the intervention. These were discussed and outlined qualitatively through the teachers and students’ focus groups.

*Contamination* referred to unintended exposure of the control group. This includes issues such as lack of blinding among participants, teachers, and outcome assessors. Other factors that may have compromised the integrity of the intervention were also considered.

### Training logbook

The training logbook was carefully designed through discussion within the research group in order to capture the aforementioned process evaluation measures. PE teachers were requested to complete training logbook for every HIIT session and sent it to the leading researcher on a weekly basis. Immediate inquiries were made to the PE teachers if there were any questions or confusion pertaining the records. The results for the training logbook record are presented in S2 File.

### Focus groups

Two focus groups with students and one focus group with teachers were conducted within one week upon completion of the PRO-HIIT intervention. Five (two girls, three boys) and four (2 girls, 2 boys) participants from two of the intervention classes consented to participate. These nine students included four peer-coaches and five randomly selected volunteers. All three PE teachers who delivered the HIIT sessions agreed to be involved in the teachers’ focus group. However, one PE teacher was not able to attend the focus group due to reasons unrelated to the study. A semi-structured design was adopted to facilitate discussion during the focus groups. The questions/topics for the focus groups (S3 File) were structured based on the MRC guidance (e.g., barriers and facilitators). The focus group sessions were conducted by the first author (YL) and recorded using a smartphone (iPhone SE, USA).

### Data analysis

The current study employed a convergent parallel mixed methods design. Quantitative data were collected for reach, recruitment and retention, dose delivered, fidelity, dose received and response. Data were analysed using IBM SPSS Statistics for Windows (SPSS 28.0; IBM Corporation, Armonk, NY, USA) unless specified. Qualitative data were collected for adaptation, response, dose received, unintended consequence, barriers and facilitators, and contamination. The focus group recordings were transcribed verbatim in Chinese and were translated to English by the first author. A second author (MHL) checked the transcribed data for completeness and accuracy. Transcripts were analysed using thematic analysis following a six-phase guidance (i.e., adaptation, response, barriers, facilitators, and contamination). The coding was initially deductive based on the MRC guidance, followed by an inductive process directed by the content of the data [25]. To maximise consistency and completeness, two authors (YL and SLD) independently conducted the thematic analysis using NVivo software (Version 15) and discussed their findings until consensus was reached. Integration of quantitative and qualitative findings occurred during the interpretation stage. Areas of convergence, complementarity, and divergence between the quantitative and qualitative findings were jointly interpreted to inform the overall conclusions of the process evaluation.

## Results

### Quantitative data

#### Reach.

The first school we contacted agreed to participate in the intervention, which contained sixteen year seven classes and five PE teachers. Three PE teachers and eight classes, totalling 372 students volunteered to take part in the study.

#### Recruitment and retention.

Of the 372 participants, three of them were excluded from taking part due to health concerns. The remaining 369 participants (13.1 ± 0.3 years, 221 males) were included in the intervention. The retention rate at T2 was 100%, despite some participants missing certain measurements or HIIT sessions for reasons such as illness or academic commitment. However, fifteen participants were lost at T3 due to school transfer (n = 7) and missed outcome measurements (n = 8), resulting in a retention rate of 96%.

#### Dose delivered.

**Fig 1:**
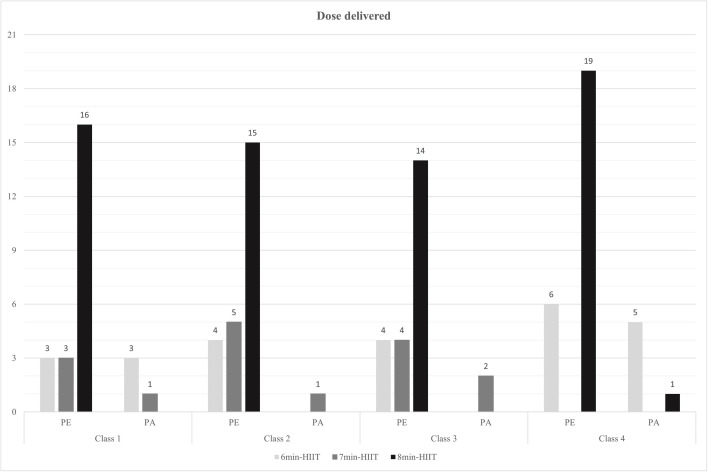
High-intensity interval training sessions delivered in physical education and physical activity lessons by intervention classes. PE, physical education; PA, physical activity; HIIT, high-intensity interval training.

Dose delivered in the PE and PA lessons at class level is presented in Fig 1. The intervention was designed to deliver HIIT sessions 5 times (3 PE + 2 PA) per week for 12 weeks, resulting in a total of 60 HIIT sessions per intervention class. However, only 26, 25, 24 and 31 sessions were delivered in the four intervention classes, respectively. Reasons for missed HIIT sessions included cancelation of PA lessons, rain, PE teachers not available, national holidays, and school events. Of note, HIIT sessions in PA lessons were all cancelled from the fifth intervention week onwards for a school-wide sports event.

#### Fidelity.

**Fig 2:**
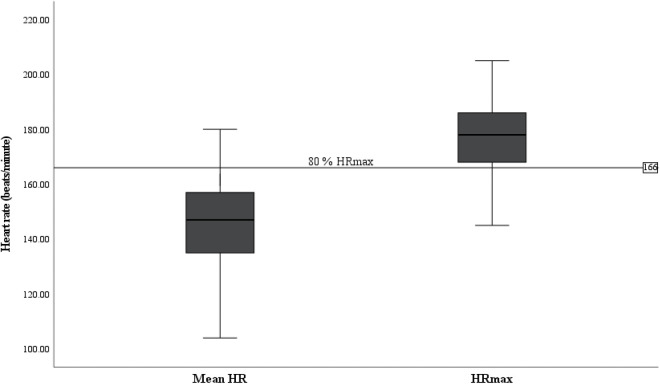
Mean and peak heart rate achieved during the high-intensity interval training sessions. HRmax, maximum heart rate; HR, heart rate.

**Fig 3:**
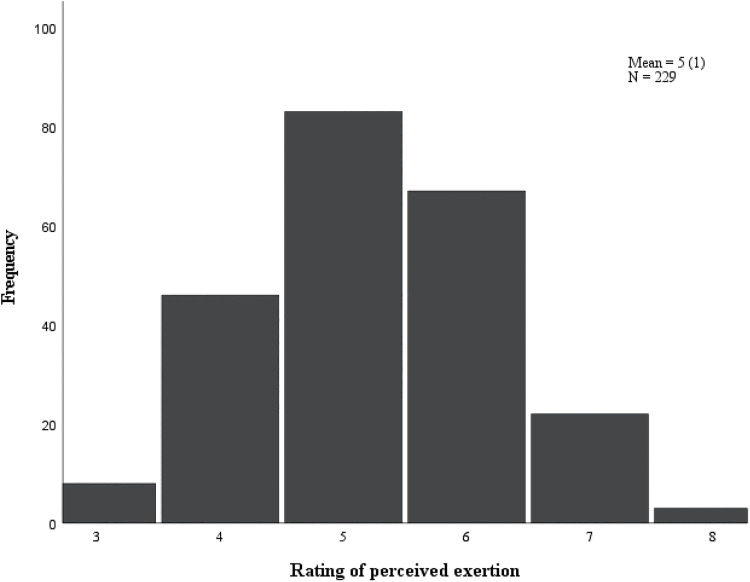
Sessional rating of perceived exertion across the high-intensity interval training.

The results for HR and RPE measures are detailed in Fig 2 and Fig 3, respectively. In total, HR measures from 127 individuals were observed across 13 HIIT sessions and RPE measures were collected from 225 individuals across 5 HIIT sessions. The average HR was 146 ± 17 beats per minute, corresponding to 71% HR_max_. Among these individual measures, 18% (n = 23) of them meeting the 80% HR_max_ threshold. The HR peak achieved by these individuals were also observed, with a mean of 175 ± 17 beats per minute (85% HR_max_), and 79% of them meeting the HIIT threshold. The average sessional RPE was 5 ± 1, which ranged from 3 to 8. The extent to which the SAAFE principles complied is presented in Table 1. Overall, compliance to the Active, Fair, and Enjoyable principles was moderate to high, whereas compliance to the Supportive and Autonomous principles were either low or unknown.

### Dose received

**Fig 4:**
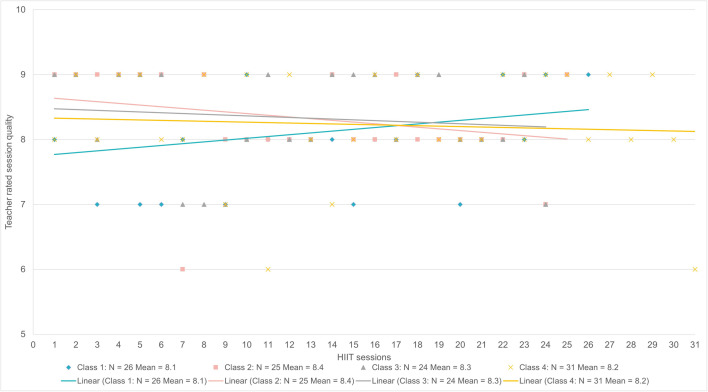
Teachers’ rating on the quality of the high-intensity interval training sessions throughout the intervention.

A total of 11, 17, 21, and 19 absences were reported for the HIIT sessions in the four intervention classes, corresponding to a dose received (attendance) rate of 99.1%, 98.5%, 98.1% and 98.6%, respectively. The reasons for absences were illness and academic commitments. The distribution and mean quality score for the four intervention classes is illustrated in Fig 4.

### Response

**Fig 5:**
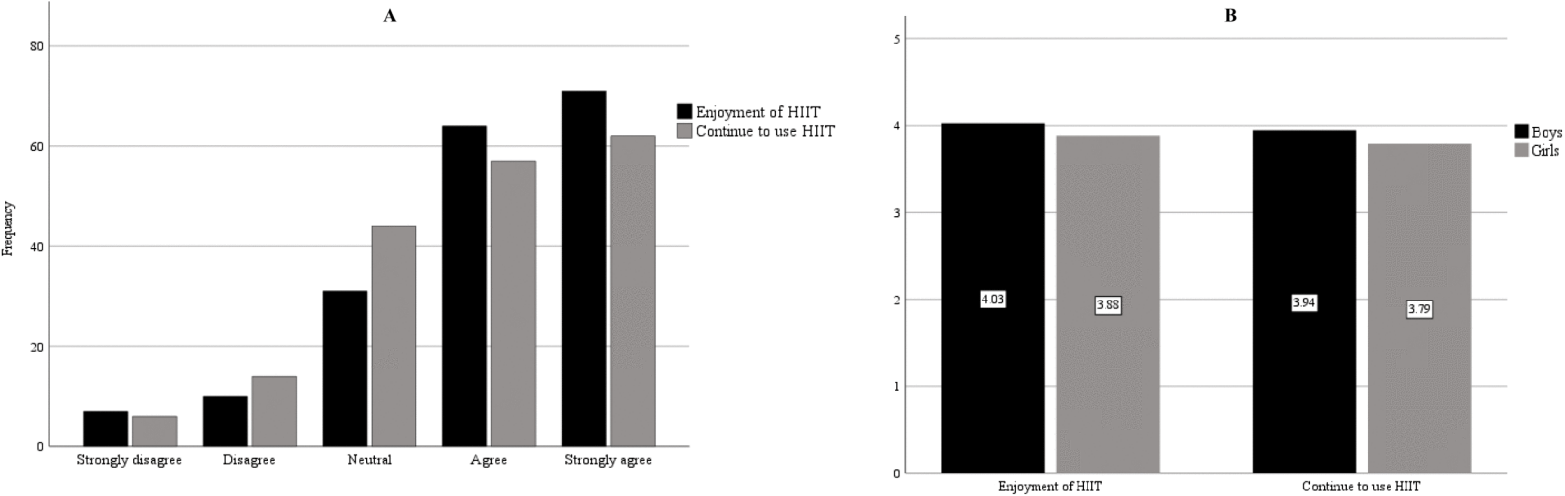
Participants’ response on enjoyment and continue to use of high-intensity interval training (A) and comparison of scores between boys and girls (B).

Fig 5 illustrates participants’ response to the HIIT programme (A) and the comparison of scores between boys and girls (B). Most participants in the intervention group rated the HIIT intervention as enjoyable or somewhat enjoyable (74%), with a mean enjoyment score of 4.0 out of a 5-point scale. Similarly, most participants expected to continue to use the HIIT protocol in the future (65%), with an average score of 3.8 on a 5-point scale. In addition, no sex differences were noted for the two scales (*p* = 0.257 and 0.769 for enjoyment and continue to use HIIT, respectively). The three PE teachers gave average ratings of 4.7 and 4.3 for liking and willingness to continue using HIIT.

### Qualitative data

#### Adaptation.

Across the intervention, several adaptations were made, as recorded in the training logbook and through the weekly communications. To account for student acceptability, session length was adjusted at the teachers’ discretion. In addition, the 10-minute HIIT sessions were shortened to 8-minute in length. Furthermore, to facilitate implementation, the option to design the HIIT sessions in the final three weeks were cancelled by the PE teachers.

“*One thing I should mention is that we think 6 to 8 minutes is an achievable duration, while we think 10 minutes may too long. We tried to deliver the 10 minutes sessions. However, the quality would start to drop after 6 to 8 minutes. Additionally, we have lesson plans for each PE lesson, and the student may be too tired for the rest of the class if HIIT sessions too long*” (Teacher B).

#### Dose received.

Both students and teachers reported that there was a small proportion of students who did not fully engage in the HIIT sessions.

*“All of them were doing the exercises, but it depends in terms of how much effort was paid. You always got a small proportion of students not fully engaged, as they always do, not just during the HIIT sessions”* (Teacher A).

*“Some classmates tried their very best, while a small portion of them were lazy. I feel like at least 70% percent of them did it very hard”* (Student focus group 1, student A).

In addition, the dose received may have diminished when delivered without supervision.

*“They liked to exaggerate those movements and sometimes, they missed some of the movements when they should. Especially if there was a lack of supervision or for those under-supervised (those boys in the last row)”* (Student focus group 2, student A).

#### Response.

Participants generally satisfied with the PRO-HIIT intervention. For example, the students thought that the intervention added variety and challenges to their traditional PE lessons.

“*The intervention added more elements to our PE classes, making it more dynamic*” (Student focus group 2, student D).

Similarly, teachers expressed positive responses to the PRO-HIIT intervention, noting that it caused no extra burden to their work.

“*You’ve made everything clear. We just did everything you required, and it didn’t increase our workload at all*” (Teacher B).

Most students liked the outcome measurements, which is in agreement with the teachers, who mentioned that the outcome measurements, conducted during PE lessons, had no impact on their curriculum goals.

“*I liked the executive function tasks, it was interesting. The colour-shape switch tests were very challenging*” (Student focus group 2, student B).

“*Some of those tests were taken place on the raining days, during which the PE would have been cancelled. In addition, those tests such as 20m-SRT were very high intensity exercises. There’s no problem for taking 20m-SRT as the content of a PE lesson*” (Teacher B).

However, one student complained that too many PE classes were used for outcome measurements, especially since the outcomes were repetitively measured.

“*I felt like too many PE classes were used. Perhaps, the tests could be more efficient, and administered in a faster manner, so we could have more time remain for playing. In addition, I don’t understand why the outcomes were measured for several times, especially those measurements like height and weight*” (Student focus group 1, student C).

In addition, students did not want the HIIT sessions to last too long, and they did not like performing HIIT sessions during PA lessons.

“*We did not want to continuously do exercises for too long. I think maximum of 6-8 minutes.*” (Student focus group 2, student A).

“*We didn’t like the PE teacher who came to ask us to do that (HIIT in PA lessons), we all felt like why we should listen to him. The HIIT session was just followed by a 10-minute running, during which I was very tired.*” (Student focus group 2, student B)

Moreover, one female student expressed that girls may not enjoy the HIIT workouts as much as boys.

“*The exercises should be suitable for both sexes. I found like the exercises we currently have were more enjoyed by boys*” (Student focus group 1, student D).

Surprisingly, the teachers disagreed that the participants were motivated by the prize (souvenir) draw. Instead, they believed that prizes should not be used as the motivation for engaging in exercise.

“*Many of the students may have forgotten it overtime (prize). Prize as motivation does not last long. Additionally, I personally believe that having to rely on prize to motivate students from doing exercise is a failure as a PE teacher*” (Teacher A).

Despite positive response to the HIIT intervention, the teachers had low confidence in the efficacy of HIIT delivered over such short HIIT sessions and intervention duration.

“*The exercises were more of improving students’ small muscle groups, agility as well as coordination. The effectiveness may take longer to be noticeable*. *For example, perhaps we keep performing it for the whole three years during the secondary school*” (Teacher A).

### Unintended consequence

No intervention related injuries happened over the intervention period.

### Barriers

***Competing priorities.*** From the fifth intervention week onwards, HIIT sessions in PA lessons were disrupted owing to a school-wide sports event, contributing to the lower dose delivered than planned. ***Severe weather.*** Both hot weather and rain threatened the implementation of the PRO-HIIT intervention. On hot days, session quality declined, while rainy days led to cancellation of PE lessons. ***Lack of sleep.*** Students reported that lack of adequate sleep negatively impacted their willingness to participate in HIIT sessions. ***Repetition of HIIT workouts.*** Limited to the number of HIIT workouts available, participants had to repeat exercises within and across sessions. This repetition led to feelings of monotony, as one student stated:

“*I felt like we repeated a lot of the exercises, which was kind of boring to me*” (Student focus group 2, student C).

***RPE administration.*** Collecting sessional RPE scores proved to be more challenging than anticipated.

“*I found measuring the RPE is complicated. I asked the peer-coaches to do this for me while I was teaching. This could be a major interrupt for the rest of the class, and it was unfair for the peer-coaches as they may miss the rest of the class.*” (Teacher A).

#### Facilitators.

***Space efficiency.*** The teachers appreciated that HIIT could be performed without equipment and in limited space, which is particularly helpful considering the scale of Chinese schools and classes.

“*One advantage of HIIT is that it does not require much space. You know we have quite a lot of PE lessons were taking place simultaneously at times. Therefore, we only have limited space for PE.*” (Teacher A).

***Imparting knowledge.*** The teachers valued the HIIT intervention for integrating sports and fitness related knowledge into their PE lessons.

“*The outcome measurement itself is a very good opportunity for imparting knowledge for sports and health.*” (Teacher A).

***Peer coaches.*** Both the students and teachers valued the significant contribution of peer coaches in facilitating the intervention implementation.

“*I think they (peer coaches) did a very good job. I mean they could perform those exercises very professionally and know exactly what the next move was*” (Student focus group 2, student B).

“*They’ve been very helpful. They helped to take the attendance every single lesson and always remind others to focus on what they should do.*” (Teacher B).

Apart from the above facilitators, students also enjoyed the ***music*** played during the HIIT sessions, and they felt motivated when their ***PE teachers were involved.***

“*Sometimes our PE teacher did the exercises with us, and I felt many of us were motivated*” (Student focus group 1, student D).

#### Contamination.

Whereas the outcome assessors were blinded to the intervention and control group, the students and PE teachers were not. The teachers reported that the intensity of HIIT was deemed higher than the traditional warm-up.

“*The HIIT group was like to perform an extra intensive dose of fitness training, while the control was just warmed up. To me, the exercise intensity in HIIT group was definitely higher compared to control*” (Teacher B).

## Discussion

The current study serves as a process evaluation to gain a deeper understanding of the findings of the PRO-HIIT intervention. Overall, the intervention demonstrated successful implementation in terms of reach, recruitment, retention, and fidelity, although the dose delivered was lower than planned. The HIIT sessions were well received, with positive responses from both teachers and students, and no injury reported over the intervention period.

Unlike studies conducted in some other countries [24,26], the current study, in line with Li et al [27], showed that Chinese schools are generally receptive to PA-related interventions and are unlikely to drop out once committed. This mirrors the fact that both teachers and students were generally satisfied with the PRO-HIIT intervention. In terms of intervention fidelity, the mean HR reached 71% of HR_max_, and the mean peak HR achieved 85% HR_max_. This aligns with findings of Kennedy et al. [19], who reported similar exercise intensities (70% and 82% HR_max_ for mean and peak HR) using bodyweight resistance-based HIIT. While the intensity achieved in our study might be slightly lower than prescribed (i.e., ≥ 80% HR_max_), it should be considered a meaningful achievement for a school-based, peer-led intervention, which is likely sufficient to confer meaningful health benefits for children and adolescents [28,29]. Considering the limitation of HR as a standalone metric for monitoring HIIT [20], sessional RPE was incorporated in the PRO-HIIT intervention to better capture HIIT intensity. The average sessional RPE reported was 5, ranging from 3 to 8. This finding is supported by Liu et al. [20], who recommended using RPE = 5 for prescribing HIIT, despite their calibration was conducted through laboratory data using running- and cycling-based protocols. Similarly, in a school-based HIIT intervention by Duncombe et al. [22], they reported a mean sessional RPE of 6, in which they also showed a slightly higher HR value (79% and 92% HR_max_ for mean and peak HR). The intensity achieved in our study is promising given the HIIT sessions were led by peer coaches rather than PE teachers. Nevertheless, it is worth noting that both HR and RPE were only partially collected from two of the intervention classes in PE lessons only due to limited financial and human resources. While we did not measure intensity in the PA lessons, it might be slightly lower based on the qualitative findings that participants did not like HIIT to be embedded in PA lessons and there was a lack of supervision during those sessions. Nonetheless, considering that only a small number of doses were delivered in PA lessons (4, 1, 2, and 6 sessions, respectively) at merely the initial four weeks of the intervention, the impact on intervention effectiveness should be trivial.

Several strategies were utilised to ensure the intervention was delivered in a supportive, active, autonomous, fair, and enjoyable manner. More than half of these strategies were applied and with medium to high compliance as shown in Table 1. To promote autonomy, the intended intervention included three weeks where student could design their own HIIT sessions, but this was not achieved as planned. This is because the design process entailed an additional session led by the head teacher prior to the sessions being performed, which was no longer feasible due to time constraints and the increased workload. This change highlighted the real-world challenges that prevent the ideal conditions envisioned in the intervention planning from being fully realised. The interaction was limited primarily between the researchers and PE teachers, whereas successful implementation of a complex intervention may require support across the school system, including head teachers, school leaders and parents [30].

In addition, participants were encouraged to make up missed HIIT sessions during the breaks or lunch time, which turned out to be impractical as no participants actually followed through. This highlights the trade-off between promoting student autonomy and maintaining intervention effectiveness, a challenge previously discussed in the literature [31]. It should be noted that the peer coaches did not receive training in the SAAFE principles during the training workshop, and we did not conduct on-site observations to evaluate the implementation of the principles in practice among PE teachers or researcher assistants (e.g., encourage mutual support or minimise controlling language). Therefore, the compliance to some of these principles remains unknown or low, which should not be overlooked in future investigations.

To enhance the likelihood of programme success, a certain degree of intervention flexibility should be expected and permitted [19,24]. In the current study, the 10-minute HIIT sessions were shortened to 8 minutes due to logistic constraints (e.g., accommodate the requirements of the PE curriculum) and to prevent declines in participants’ effort over extended HIIT durations. This aligns with findings of Kennedy et al. [19], who found that 8-minute HIIT sessions were the most popular in the Burn 2 Learn programme. Considering that this adaptation may lead to a ‘penalty’ in the effectiveness of the PRO-HIIT intervention [32], the work-to-rest ratio was, accordingly, increased during the final four weeks to offset the shorter session duration. Despite this change, feedback from the focus groups indicated that students did not find the adjustment overly challenging, potentially due to improvements in physical fitness. Balancing the rigid implementation of study protocols with the needs of stakeholders is critical to the success of interventions [26]. In the case of the PRO-HIIT intervention, the adaptations were made through mutual agreement between the research team and the PE teachers, underscoring the pivotal role of regular, seamless, and constructive communication. Since a one-size-fits-all approach is not always effective, future studies should consider such adaptations in their study design to better address specific needs.

Only one intervention class achieved the expected minimum of 30 sessions as planned in our study protocol [14]. This is primarily due to the suspension of HIIT sessions in PA lessons from the fifth week onwards to accommodate a school-wide sports event, leading to only 13.5% of prescribed does being delivered in PA lessons as compared to 64.6% in PE lessons. Other factors contributed to missing the sessions were rain, availability of PE teachers, and a national holiday. While the dose delivered may not be satisfactory, clearly, it was the inevitable factors that hindered the intervention delivery. In other words, the HIIT sessions could be delivered unless PE lessons were interrupted, showing that the schoolteachers were generally able to provide the agreed-upon dosage of HIIT [19,24]. On the contrary, the HIIT sessions were well received by the participants, as revealed by high adherence rates (all > 98%) and session quality (Fig 4), which was also corroborated by both students and teachers who reported that only a small proportion of students did not fully engage in the HIIT sessions. This finding is inconsistent with previous school-based HIIT interventions [24,33,34], revealing declines in session adherence and/or engagement over time, which likely stem from reduced motivation towards HIIT. However, in the PRO-HIIT study, participants valued the benefits of HIIT for its variety of movements. They found the traditional warm-ups were monotonous and ineffective by queuing up and running in structured order and pace. According to self-determination theory, the benefits of HIIT were personally recognised (identified regulation), while the traditional warm-ups were driven by external pressures, such as teachers (external regulation), supporting the sustained adherence and engagement observed in our study [35,36].

### Future suggestions

Based on the process evaluation for the PRO-HIIT intervention, several take-home messages are worth highlighting to inform future practices. First, future interventions should have a through consideration on time commitment of both the intervention itself and the associated outcome measurements and try to find a balance between flexibility and intervention fidelity. This is crucial, as failing to address participants’ and stakeholders’ needs could undermine implementation commitments, leading it to falter, stagnate or even drop out [26]. Additionally, future interventions should incorporate a wider variety of HIIT workouts as well as take gender difference into consideration. Although the PRO-HIIT intervention offered more than ten different workouts, focus group findings suggested that this variety was insufficient across the twelve-week programme. Consequently, student engagement or motivation may have been somewhat reduced as the providing of variety is key to the enjoyment and motivation for engaging in HIIT [37]. Interestingly, this finding appears to contrast with the high retention rate, which is likely influenced by social factors, such as social conformity or the desire to perform well in front of teachers. In addition, one girl has suggested to consider gender differences in the design of future exercises – an insight that merits attention. Girls were frequently reported to engage in less PA as compared to boys [38]. Actions should be taken to encourage their compliance and engagement. Otherwise, there is a risk that they may be disengaged or less motivated than boys, which could subsequently lead to intervention-generated PA inequity [39,40]. Furthermore, future interventions are recommended to adopt peer coaches to assist intervention delivery – a strategy with the potential to alleviate PE teachers’ workload while fostering autonomy among participants. This approach is particularly promising for future PA interventions that rely on non-PE staff members, as they typically lack confidence in delivering PA programmes effectively [41]. Moreover, unlike previous studies [11], it seems using prize as incentives to promote participation and engagement may not a particularly effective strategy in the PRO-HIIT intervention and hence are not recommended. The teachers believed that incentives provide only short-term motivation and concerned about relying on extrinsic rewards to influence students’ motivation for PA participation, an proposition similar to that of Mitchell et al. [42]. Future studies could focus on identifying incentives that cater participants’ needs while, more importantly, prioritising strategies to foster intrinsic motivation to ensure sustainable engagement and adherence.

### Strength and limitations

The current study utilised a convergent parallel mixed methods approach, guided by the MRC framework to give a complete picture of the process evaluation. The integration of quantitative and qualitative data enabled triangulation across process evaluation domains and reduced the likelihood of incomplete reporting or biased interpretation. However, several limitations should be acknowledged. First, the students and teachers were not blinded to the intervention groups and the session intensity were only measured in a sub-sample of participants and in PE lessons only. This could potentially contaminate or obscure our research findings. Second, several process evaluation outcomes were assessed using subjective measures (e.g., session quality scores), which may have been susceptible to social desirability bias. Third, the qualitative sample comprised only nine students across two focus groups and two PE teachers who participated in individual interviews. Although these data provided valuable insights into participants’ experiences and perceptions of the intervention, the sample size may have limited the breadth and diversity of perspectives captured. The qualitative component was designed to complement the quantitative process evaluation rather than to achieve exhaustive thematic saturation [43]. Therefore, the qualitative findings should be interpreted as an exploratory thematic overview of key implementation experiences, barriers, facilitators, and perceived impacts within this context.

## Conclusion

The present study provides valuable insights into the “how” and “why” the PRO-HIIT intervention works or not within a Chinese secondary school setting, utilising a convergent parallel mixed methods process evaluation. Overall, the PRO-HIIT intervention demonstrated favourable reach, retention, and dose received as indicated by the positive responses from both teachers and students. However, the dose delivered fell slightly below the predefined threshold mainly due to uncontrollable competing priorities, and implementation fidelity was only partially achieved as intended. Our study demonstrated the potential for incorporating HIIT in existing PA curriculum time to engage Chinese children and adolescents in vigorous PA. As a reflection, the implementation of the PRO-HIIT intervention may have come at the expense of students’ autonomy. Future studies should aim to better balance implementation fidelity and autonomy support and seek to foster PA motivation through intrinsic means. Given the exploratory nature of the qualitative component, further research involving larger and more diverse qualitative samples is warranted to confirm and extend the implementation insights identified in the present study.

## Supporting information

S1 File High-intensity interval training session design and workouts for the PRO-HIIT intervention. (DOCX)

S2 File Details for the teachers’ training logbook record. (DOCX)

S3 File Teachers and students’ focus groups materials. (DOCX)
