# Supplementary material for: Understanding the “how” and “why”: A mixed methods process evaluation for the PRO-HIIT intervention
Source: PLoS One. 2026 Jun 30;21(6):e0352772. doi: 10.1371/journal.pone.0352772 (PMC13318028; doi:10.1371/journal.pone.0352772)
Supplement: S1 File — (DOCX) [file pone.0352772.s001.docx]

| Supplementary File 1. High-intensity interval training session design and workouts for the PRO-HIIT intervention.   \|  \| Week 1 – 3 \| Week 4 – 6 \| Week 7 – 9 \| Week 10 – 12 \| \| --- \| --- \| --- \| --- \| --- \| \| Session design \| 6-minute HIIT  1-2-minute: 10 s: 20 s  3-4-minute: 15 s: 15 s  5-6-minute: 20 s: 10 s \| 8-minute HIIT  1-2-minute: 10 s: 20 s  3-5-minute: 15 s: 15 s  6-8-minute: 20 s: 10 s \| 10-minute HIIT  1-2-minute: 10 s: 20 s  3-6-minute: 15 s: 15 s  7-10-minute: 20 s: 10 s \| 10-minute HIIT  1-5-minute: 15 s: 15 s  6-10-minute: 20 s: 10 s \| \| HIIT exercises \| Jumping jacks, high knees, burpees, butt kicks, mountain climbers, lunge jumps (2/workouts x 6) \| Running on the spot, squat jumps, high knees, star jumps, burpees, lunge jumps, mountain climbers, push ups (2/workouts x 8) \| Running on the spot, rocket jumps, high knees, push ups, burpees, boxing, mountain climbers, butt kicks, jumping jacks, side to side skiers (2/workouts x 10) \| Students select HIIT workouts from an exercise pool (20 workouts, maximum repeat 4 times per workout) \| \| Exercise pool:  Running on the spot  squat jumps  jumping jacks  high knees  burpees  butt kicks  mountain climbers  lunge jumps, star jumps  lunge jumps  rocket jumps  side to side skiers  push ups  boxing  (n = 14) \| \| \| \| \| |
| --- | --- | --- | --- | --- | --- | --- | --- | --- | --- | --- | --- | --- | --- | --- | --- | --- | --- | --- | --- | --- |
