# Supplementary material for: Understanding the “how” and “why”: A mixed methods process evaluation for the PRO-HIIT intervention
Source: PLoS One. 2026 Jun 30;21(6):e0352772. doi: 10.1371/journal.pone.0352772 (PMC13318028; doi:10.1371/journal.pone.0352772)
Supplement: S2 File — (DOCX) [file pone.0352772.s002.docx]

Supplementary File 2. Details for the teachers’ training logbook record.

| **Teacher's training logbook record** | | | | | | |
| --- | --- | --- | --- | --- | --- | --- |
|  |  | Classes | HIIT (min) | Attendance (presented as ID + reason) | Session quality | Others (e.g., injury, adaptation) |
| Intervention week 1 | Monday | 1 | Session not performed due to raining | | | |
|  |  | 2 |  |  |  |  |
|  |  | 3 |  |  |  |  |
|  |  | 4 |  |  |  |  |
|  | Tuesday | 1 | Session not performed due to a school event | | | |
|  |  | 2 |  |  |  |  |
|  |  | 3 |  |  |  |  |
|  |  | 4 |  |  |  |  |
|  | Wednesday | 1 | PA lesson cancelled due to a school event | | | |
|  |  | 2 |  |  |  |  |
|  |  | 3 |  |  |  |  |
|  |  | 4 | 6-min HIIT |  | 8 | None |
|  | Thursday | 1 | School event | | | |
|  |  | 2 | 6-min HIIT |  | 9 | None |
|  |  | 3 | PA lesson cancelled | | | |
|  |  | 4 | 6-min HIIT | 22, academic commitment | 9 | None |
|  | Friday | 1 | PA lesson cancelled | | | |
|  |  | 2 |  |  |  |  |
|  |  | 3 | 6-min HIIT | 13, 27, illness | 9 | None |
|  |  | 4 | 6-min HIIT |  | 8 | None |
|  | | | | | | |
| Intervention week 2 | Monday | 1 | 6-min HIIT | 31, illness | 8 | None |
|  |  | 2 | PE teacher not available | | | |
|  |  | 3 |  |  |  |  |
|  |  | 4 | 6-min HIIT* |  | 9 | None |
|  | Tuesday | 1 | 6-min HIIT | 25, academic | 9 | None |
|  |  | 2 | PE teacher not available | | | |
|  |  | 3 |  |  |  |  |
|  |  | 4 | 6-min HIIT | 31, illness | 9 | None |
|  | Wednesday | 1 | 6-min HIIT |  | 7 | None |
|  |  | 2 | PA lesson cancelled due to competing interest | | | |
|  |  | 3 |  |  |  |  |
|  |  | 4 | 6-min HIIT | 31, illness | 8 | None |
|  | Thursday | 1 | National holiday | | | |
|  |  | 2 |  |  |  |  |
|  |  | 3 |  |  |  |  |
|  |  | 4 |  |  |  |  |
|  | Friday | 1 |  |  |  |  |
|  |  | 2 |  |  |  |  |
|  |  | 3 |  |  |  |  |
|  |  | 4 |  |  |  |  |
|  |  |  |  |  |  |  |
| Intervention week 3 | Monday | 1 | Session not performed due to a school event | | | |
|  |  | 2 | 6-min HIIT | 40, illness | 9 | None |
|  |  | 3 | 6-min HIIT | 46, illness | 9 | None |
|  |  | 4 | 6-min HIIT | 9, illness | 8 | None |
|  | Tuesday | 1 | 6-min HIIT |  | 9 | None |
|  |  | 2 | 6-min HIIT | 40, illness | 9 | None |
|  |  | 3 | 6-min HIIT | 46, illness | 8 | None |
|  |  | 4 | 6-min HIIT | 9, illness | 9 | None |
|  | Wednesday | 1 | 6-min HIIT |  | 7 | None |
|  |  | 2 | PA lesson cancelled | | | |
|  |  | 3 |  |  |  |  |
|  |  | 4 | 6-min HIIT | 9, illness | 7 | None |
|  | Thursday | 1 | School event | | | |
|  |  | 2 | 6-min HIIT | 40, illness | 9 | None |
|  |  | 3 | PA lesson cancelled | | | |
|  |  | 4 | 6-min HIIT* |  | 9 | None |
|  | Friday | 1 | 6-min HIIT |  | 7 | None |
|  |  | 2 | PA lesson cancelled | | | |
|  |  | 3 | 6-min HIIT | 46, illness; 14, academic | 9 | None |
|  |  | 4 | 6-min HIIT |  | 6 | None |
|  |  |  |  |  |  |  |
| Intervention week 4 | Monday | 1 | 7-min HIIT | 16, academic | 8 | Class 1, 2, and 3 performed 7-minute  of the 8-minute HIIT |
|  |  | 2 | 7-min HIIT |  | 9 |  |
|  |  | 3 | 7-min HIIT | 32, illness | 9 |  |
|  |  | 4 | 8-min HIIT |  | 9 | None |
|  | Tuesday | 1 | 7-min HIIT |  | 9 | None |
|  |  | 2 | 7-min HIIT | 39, illness | 9 | None |
|  |  | 3 | 7-min HIIT | 13, 32, illness | 9 | None |
|  |  | 4 | 8-min HIIT* |  | 8 | None |
|  | Wednesday | 1 | 7-min HIIT |  | 7 | None |
|  |  | 2 | 7-min HIIT |  | 6 | None |
|  |  | 3 | 7-min HIIT |  | 7 | None |
|  |  | 4 | 8-min HIIT |  | 7 | None |
|  | Thursday | 1 | 7-min HIIT |  | 9 | None |
|  |  | 2 | 7-min HIIT |  | 9 | None |
|  |  | 3 | 7-min HIIT |  | 7 | None |
|  |  | 4 | 8-min HIIT |  | 8 | None |
|  | Friday | 1 | Session not performed due to raining | | | |
|  |  | 2 |  |  |  |  |
|  |  | 3 |  |  |  |  |
|  |  | 4 |  |  |  |  |
|  |  |  |  |  |  |  |
| Intervention week 5 | Monday | 1 | Session not performed due to raining | | | |
|  |  | 2 |  |  |  |  |
|  |  | 3 |  |  |  |  |
|  |  | 4 |  |  |  |  |
|  | Tuesday | 1 | 8-min HIIT |  | 8 | None |
|  |  | 2 | 7-min HIIT |  | 8 | Class 2 and 3 continued to perform  7-minute. |
|  |  | 3 | 7-min HIIT | 9, academic; 35, 40 illness | 7 |  |
|  |  | 4 | 8-min HIIT* |  | 9 |  |
|  | Wednesday | 1 | All HIIT session in PA classes were cancelled due to a school-level sport event across the whole semester. | | | |
|  |  | 2 |  |  |  |  |
|  |  | 3 |  |  |  |  |
|  |  | 4 |  |  |  |  |
|  | Thursday | 1 | 8-min HIIT |  | 8 | None |
|  |  | 2 | 7-min HIIT |  | 8 | None |
|  |  | 3 |  |  |  |  |
|  |  | 4 | 8-min HIIT |  | 8 | None |
|  | Friday | 1 |  |  |  |  |
|  |  | 2 |  |  |  |  |
|  |  | 3 | 7-min HIIT |  | 8 | None |
|  |  | 4 |  |  |  |  |
|  |  |  |  |  |  |  |
| Intervention week 6 | Monday | 1 | 8-min HIIT |  | 8 | None |
|  |  | 2 | PE teacher not available | | | |
|  |  | 3 |  |  |  |  |
|  |  | 4 | 8-min HIIT | 31, illness | 9 | None |
|  | Tuesday | 1 | Session not performed due to raining | | | |
|  |  | 2 |  |  |  |  |
|  |  | 3 |  |  |  |  |
|  |  | 4 |  |  |  |  |
|  | Wednesday | 1 | National holidays | | | |
|  |  | 2 |  |  |  |  |
|  |  | 3 |  |  |  |  |
|  |  | 4 |  |  |  |  |
|  | Thursday | 1 |  |  |  |  |
|  |  | 2 |  |  |  |  |
|  |  | 3 |  |  |  |  |
|  |  | 4 |  |  |  |  |
|  | Friday | 1 |  |  |  |  |
|  |  | 2 |  |  |  |  |
|  |  | 3 |  |  |  |  |
|  |  | 4 |  |  |  |  |
|  |  |  |  |  |  |  |
| Intervention week 7 | Monday | 1 | Teacher not available | | | |
|  |  | 2 | 8-min HIIT | 6, 40, illness | 8 | None |
|  |  | 3 | 8-min HIIT |  | 9 | None |
|  |  | 4 | 8-min HIIT |  | 8 | None |
|  | Tuesday | 1 | 8-min HIIT |  | 8 | None |
|  |  | 2 | 8-min HIIT | 6, illness | 8 | None |
|  |  | 3 | 8-min HIIT |  | 8 | None |
|  |  | 4 | 8-min HIIT* |  | 8 | None |
|  | Wednesday | 1 |  |  |  |  |
|  |  | 2 |  |  |  |  |
|  |  | 3 |  |  |  |  |
|  |  | 4 |  |  |  |  |
|  | Thursday | 1 | 8-min HIIT |  | 7 | None |
|  |  | 2 | 8-min HIIT | 6, illness | 8 | None |
|  |  | 3 |  |  |  |  |
|  |  | 4 | 8-min HIIT |  | 8 | None |
|  | Friday | 1 |  |  |  |  |
|  |  | 2 |  |  |  |  |
|  |  | 3 | 8-min HIIT |  | 8 | None |
|  |  | 4 |  |  |  |  |
|  |  |  |  |  |  |  |
| Intervention week 8 | Monday | 1 | 8-min HIIT | 41, illness | 8 | None |
|  |  | 2 | 8-min HIIT | 40, illness | 9 | None |
|  |  | 3 | 8-min HIIT | 30, 44, illness | 9 | None |
|  |  | 4 | 8-min HIIT |  | 9 | None |
|  | Tuesday | 1 | 8-min HIIT | 41, illness | 8 | None |
|  |  | 2 | 8-min HIIT | 40, illness | 8 | None |
|  |  | 3 | 8-min HIIT | 30, 44, illness | 9 | None |
|  |  | 4 | 8-min HIIT | 37, illness | 8 | None |
|  | Wednesday | 1 |  |  |  |  |
|  |  | 2 |  |  |  |  |
|  |  | 3 |  |  |  |  |
|  |  | 4 |  |  |  |  |
|  | Thursday | 1 | 8-min HIIT |  | 9 | None |
|  |  | 2 | 8-min HIIT |  | 8 | None |
|  |  | 3 |  |  |  |  |
|  |  | 4 | 8-min HIIT |  | 9 | None |
|  | Friday | 1 |  |  |  |  |
|  |  | 2 |  |  |  |  |
|  |  | 3 | 8-min HIIT |  | 9 | None |
|  |  | 4 |  |  |  |  |
|  |  |  |  |  |  |  |
| Intervention week 9 | Monday | 1 | 8-min HIIT |  | 8 | Start from week 9,  work to rest ratio: 20s : 10s |
|  |  | 2 | 8-min HIIT | 6, 39, illness | 9 |  |
|  |  | 3 | 8-min HIIT |  | 8 |  |
|  |  | 4 | Session not performed due to a school event | | | |
|  | Tuesday | 1 | 8-min HIIT |  | 7 | None |
|  |  | 2 | 8-min HIIT | 39, 40 illness | 8 | None |
|  |  | 3 | 8-min HIIT | 35, illness | 9 | None |
|  |  | 4 | Session not performed due to a school event | | | |
|  | Wednesday | 1 |  |  |  |  |
|  |  | 2 |  |  |  |  |
|  |  | 3 |  |  |  |  |
|  |  | 4 |  |  |  |  |
|  | Thursday | 1 | Session not performed due to a school event | | | |
|  |  | 2 | 8-min HIIT |  | 8 | None |
|  |  | 3 |  |  |  |  |
|  |  | 4 | Session not performed due to a school event | | | |
|  | Friday | 1 |  |  |  |  |
|  |  | 2 |  |  |  |  |
|  |  | 3 | 8-min HIIT |  | 9 | None |
|  |  | 4 |  |  |  |  |
|  |  |  |  |  |  |  |
| Intervention week 10 | Monday | 1 | Session not performed due to raining | | | |
|  |  | 2 |  |  |  |  |
|  |  | 3 |  |  |  |  |
|  |  | 4 | 8-min HIIT | 31, 43 illness; 36, academic | 9 | None |
|  | Tuesday | 1 | 8-min HIIT |  | 8 | None |
|  |  | 2 | 8-min HIIT |  | 8 | None |
|  |  | 3 | 8-min HIIT | 13, illness | 8 | None |
|  |  | 4 | 8-min HIIT | 31, 36 illness | 8 | None |
|  | Wednesday | 1 |  |  |  |  |
|  |  | 2 |  |  |  |  |
|  |  | 3 |  |  |  |  |
|  |  | 4 |  |  |  |  |
|  | Thursday | 1 | Children's day activity | | | |
|  |  | 2 |  |  |  |  |
|  |  | 3 |  |  |  |  |
|  |  | 4 |  |  |  |  |
|  | Friday | 1 |  |  |  |  |
|  |  | 2 |  |  |  |  |
|  |  | 3 | Session not performed due to raining | | | |
|  |  | 4 |  |  |  |  |
|  |  |  |  |  |  |  |
| Intervention week 11 | Monday | 1 | Session not performed due to raining | | | |
|  |  | 2 |  |  |  |  |
|  |  | 3 |  |  |  |  |
|  |  | 4 |  |  |  |  |
|  | Tuesday | 1 | 8-min HIIT |  | 9 | None |
|  |  | 2 | 8-min HIIT |  | 8 | None |
|  |  | 3 | 8-min HIIT | 7, 14, academic | 8 | None |
|  |  | 4 | 8-min HIIT | 15, 21, illness | 9 | None |
|  | Wednesday | 1 |  |  |  |  |
|  |  | 2 |  |  |  |  |
|  |  | 3 |  |  |  |  |
|  |  | 4 |  |  |  |  |
|  | Thursday | 1 | 8-min HIIT | 28, illness | 8 | None |
|  |  | 2 | 8-min HIIT |  | 8 | None |
|  |  | 3 |  |  |  |  |
|  |  | 4 | 8-min HIIT | 36, 43 illness | 8 | None |
|  | Friday | 1 |  |  |  |  |
|  |  | 2 |  |  |  |  |
|  |  | 3 | 8-min HIIT |  | 8 | None |
|  |  | 4 |  |  |  |  |
|  |  |  |  |  |  |  |
| Intervention week 12 | Monday | 1 | 8-min HIIT | 28, academic; 32, illness | 9 | None |
|  |  | 2 | 8-min HIIT | 3, illness | 9 |  |
|  |  | 3 | 8-min HIIT | 2, academic | 9 |  |
|  |  | 4 | 8-min HIIT |  | 9 |  |
|  | Tuesday | 1 | 8-min HIIT | 32, illness | 9 | None |
|  |  | 2 | 8-min HIIT | 3, illness | 7 |  |
|  |  | 3 | 8-min HIIT |  | 7 |  |
|  |  | 4 | 8-min HIIT | 28, illness | 8 | None |
|  | Wednesday | 1 |  |  |  |  |
|  |  | 2 |  |  |  |  |
|  |  | 3 |  |  |  |  |
|  |  | 4 |  |  |  |  |
|  | Thursday | 1 | 8-min HIIT | 32, 41, illness | 9 | None |
|  |  | 2 | 8-min HIIT | 3, illness | 9 | None |
|  |  | 3 |  |  |  |  |
|  |  | 4 | 8-min HIIT | 22, illness | 6 | None |
|  | Friday | 1 |  |  |  |  |
|  |  | 2 |  |  |  |  |
|  |  | 3 | PE teacher not available | | | |
|  |  | 4 |  |  |  |  |

HIIT, high-intensity interval training; PA, physical activity; 1,2,3, and 4 in the “Classes” column represents the four intervention classes; Green colour represents high-intensity interval training sessions delivered in physical education lessons; pink colour represents high-intensity interval training sessions delivered in physical activity lessons; PE, physical education.
