# Supplementary material for: Understanding the “how” and “why”: A mixed methods process evaluation for the PRO-HIIT intervention
Source: PLoS One. 2026 Jun 30;21(6):e0352772. doi: 10.1371/journal.pone.0352772 (PMC13318028; doi:10.1371/journal.pone.0352772)
Supplement: S3 File — (DOCX) [file pone.0352772.s003.docx]

**Teachers’ focus group**

**Topics**

- 1. 1. How did you feel when delivering the HIIT workouts?
  2. a. What worked well?
  3. b. What did not work well?

2. How did you feel about delivering the intervention?

3. Do you think the peer-coaches have done a great job in leading the workouts?

- 1. 4. How did the students react to the HIIT workouts?
  2. a. What did they enjoy?
  3. b. What did they not enjoy?
  4. c. What changes, if any, did you notice over the course of the intervention with regard to the students’ engagement in the HIIT workouts?
  5. d. What differences, if any, did you notice between the students involved in the HIIT intervention and the students that were not?

5. Which workouts, if any, seemed to work better? Why do you think that is?

6. what, if any, do you think is the barrier/facilitator of the programme for incorporating in classes?

7. Are there any HIIT workouts (these specific ones or others) that you will continue to use?

8. How do you find about the outcome assessment?

9. What, if anything, could we have done differently to optimise the intervention implementation?

10. Are there any differences between HIIT sessions for the physical education lessons and physical activity lessons (e.g., quality, intensity etc.)? If yes, why?

11. Is there anything else you would like to talk about that we haven’t mentioned?

**Students’ focus group**

**Topics**

- 1. 1. How did you feel about the HIIT workouts? Did they seem hard or easy to you, and can you explain why?
  2. a. Too heavy
  3. b. Suitable
  4. c. Too simple

2. How do you like the idea of increase the duration of the HIIT sessions progressively?

3. What, if anything, could we have done differently to make you like the workouts more?

4. Are there any HIIT workouts (these specific ones or others) that you will continue to use?

5. Compared to other exercises (e.g., running or basketball), which do you like best?

6. We have measurements before the intervention started and immediately after the intervention and we are going to compare the differences at the two measurement timepoints in order to determine if your opinions on physical activity, cognition and physical fitness are improved. These measurements including the executive function tasks and questionnaires you have finished in the computer room, and also the fitness measurements, including the 20-meter shuttle run, standing long jump, body composition, handgrip, waist circumference and bone health. What do you like/dislike the outcome measures and why?

7. For peer-coaches, how do you like the experience of leading the HIIT session? Do you feel confident about it?

8. How did you like the HIIT sessions delivered by the peer-coaches?

9. Do you think you performed equally well during physical education lessons and physical activity lessons? If no, why?

10. Is there anything else you would like to talk about that we haven’t mentioned?
