# Supplementary material for: Understanding the “how” and “why”: A mixed methods process evaluation for the PRO-HIIT intervention
Source: PLoS One. 2026 Jun 30;21(6):e0352772. doi: 10.1371/journal.pone.0352772 (PMC13318028; doi:10.1371/journal.pone.0352772)
Supplement: Attachment — Submitted filename: Responses_Reviewer2_PONE-D-25-62980R1.docx [file pone.0352772.s006.docx]

19 May 2026

Dear reviewers,

**RE: REVISION, PLOS ONE, PONE-D-25-62980 “Understanding the “how” and “why”: a mixed methods process evaluation for the PRO-HIIT intervention”.**

Thank you very much again for your thorough review of our article. Similar to the revision one, we repeated your comments and provided our response accordingly. All revisions made to the manuscript have been highlighted in yellow, and the relevant revised text is repeated in this document in *italics*, where appropriate.

We look forward to hearing your thoughts on our revised manuscript.

Kind regards,

The authors

**Reviewer #1:**

Thank you very much for your response to my comments and for making certain amendments to the new version of the manuscript with the aim of accepting my suggestions. I understand that some of my suggestions were hard to accept, considering the fact that you already have extensive list of publications in which this type of training has been designed and investigated. However, I do not agree with some of your comments made. Anyway, I recommend acceptance of the manuscript as you probably would not be willing to change your mind in accordance with my proposals.

1. The control of intensity during HIIT (but real HIIT) in school-based settings is not unfeasible. I do it all the time and it works perfectly if running is the exercise modality.
2. The fact that “body-weight, multi-joint, cyclic activities are commonly adopted in school interventions and have been widely used in previous school-based HIIT research” does not mean that they are better or that they are optimal.
3. The criterium of time spent ≥90% VO2max is widely accepted criterium and it is recognized as a key physiological stimulus with physiological rationale backing it up (Odden et al., 2024; Molmen & Ronnestand, 2024). There are other references as well, of course. Other criteria are arbitrarily defined and that is why they are inferior to the one mentioned above.
Odden, I., Nymoen, L., Urianstad, T., Kristoffersen, M., Hammarström, D., Hansen, J., Mølmen, K.S., & Rønnestad, B.R. (2024). The higher the fraction of maximal oxygen uptake is during interval training, the greater is the cycling performance gain. European Journal of Sport Science, 24(11), 1583-1596.
Mølmen, K.S. & Rønnestad, B.R. (2024). A narrative review exploring advances in interval training for endurance athletes. Applied Physiology, Nutrition, and Metabolism, 49(7), 1008-1013.
4. Even though your primary aim was not to assess physiological adaptations, the training program investigated needs to be properly prescribed. If the program is not prescribed properly, then everything else is false. All results presented in your study are basically not related to HIIT, but to some other type of training.
5. Claiming that this type of HIIT (which is not HIIT) is more feasible, or only feasible, in school-setting is wrong. Real HIIT sessions, which are optimal for improvement of VO2max which is the main health-related physical fitness component, are very easily incorporated withing school settings. Studies which claim that inferior types of HIIT are more feasible and optimal are creating “noise” in the literature and offer inferior practical solutions. HIIT is HIIT. There is no school-based HIIT literature. Or there shouldn’t be school-based HIIT literature.
6. The fact that you used 220-age to assess HRmax is unacceptable for scientific publication. Check the variability in such estimation, please. I understand that estimation is more feasible, but some things need to be done properly.
7. You claim that “It is also not feasible to retain children and adolescent participants under such high exercise intensity”. It is feasible, you just need to prescribe exercise better to make it happen. That is the main purpose of being a coach or a PE teacher. And to add to that, intensity has been recognized as the key variable for health enhancement (Schwendinger et al., 2025), so trying to make students work at higher intensity, but with keeping the RPE low, is the ultimate goal in finding the right HIIT design.
Schwendinger, F., Infanger, D., Lichtenstein, E., Hinrichs, T., Knaier, R., Rowlands, A. V., & Schmidt-Trucksäss, A. (2025). Intensity or volume: the role of physical activity in longevity. European Journal of Preventive Cardiology, 32(1), 10–19.
8. The fact that Buchheit and Laursen’s paper is over a decade old does not make it obsolete. It does not change the fact that the propositions presented do not still stand. Again, saying that there is heterogeneity in HIIT studies is making “noise” and creating confusion. However, the main issue is that no optimal practical solutions are provided when poor program designs are labeled as HIIT.
9. The ≥90% VO2max/HRmax should be accepted as the main criterium and, as I pointed out earlier, we need to find the exercise design in which time ≥90% HRmax would be maximized but lowest levels of RPE and blood lactate and highest level of enjoyment and affect. Choosing the right exercises is the first step. And the choice should not be left to students to decide.

**Response:**

We greatly appreciate your acceptance of our manuscript despite our differences on several points. To our knowledge, no publication has claimed or argued that only running and cycling should be classified as HIIT, or that an intensity of ≤90% VO₂max is insufficient for classification as HIIT. In addition, there is not one single marker of intensity, with HR, RPE, and affect all widely used in the literature. Likewise, the presence of school-based HIIT literature has not been disputed. As you may be also aware, numerous HIIT reviews have reported that resistance- and game-based HIIT are commonly adopted exercise modalities and are becoming increasingly popular as they offer further flexibility and adaptability to school-based programmes, compared to traditional running- or cycling-based HIIT. This evolution has contributed to the implementation and scalability of school-based interventions and are important from a public health perspective.

Indeed, the presence of these exercise modalities does not imply that they are superior or optimal, they just provide alternative options. Therefore, unless and until scientific evidence demonstrates, this area of research, including the current study, should not be described as “noise,” but rather as part of the continued development of contemporary HIIT literature.

Despite these differing perspectives we look forward to future scientific debate on this topic.

Regarding your comments on the estimation of HRmax, we fully acknowledge the limitations of using the “220-age” equation. However, as stated in the manuscript, resource limitations prevented us from objectively measuring HRmax for each participant. We also recognize that there are other alternative estimation formulas that may provide improved accuracy, such as 208–0.7*age. Nevertheless, because we used Polar devices, which apply the 220-age equation to determine HR zones (e.g., Zone 4 represents 81–90% HRmax; see [https://www.polar.com/en/guide/calculate-max-heart rate?srsltid=AfmBOorCHY7B_AJAbmBk_5CQ0vm27Fjy-LrCXYtRi5bmoryobQvQ6nsq](https://www.polar.com/en/guide/calculate-max-heart%20rate?srsltid=AfmBOorCHY7B_AJAbmBk_5CQ0vm27Fjy-LrCXYtRi5bmoryobQvQ6nsq)), we considered it appropriate to maintain consistency with this calculation method.

**Reviewer #2:**

- Reviewer #2: Dear Authors,
  Thank you for your revised manuscript that shows substantive efforts made to address the methodological and interpretative concerns raised during the previous peer review process. The clarification of the high-intensity interval training definition within a school-based context, as opposed to a strictly controlled laboratory environment, significantly elevates the practical relevance of the work. Furthermore, the improved reporting of the heart rate intensity thresholds and the recategorization of the school sports event as a contextual barrier rather than an unintended consequence enhance the precision and transparency of the process evaluation narrative. The expanded limitations section appropriately contextualizes the small qualitative sample size, ensuring the findings are interpreted with suitable academic caution.

**Response:**

Thank you very much for your positive words; your insightful suggestions have improved the manuscript.

- Addressing the methodological descriptions of the intervention delivery, the decision to omit the SAAFE principles from the peer coach training is adequately justified in the rebuttal. However, this creates a minor gap in the fidelity assessment narrative that warrants brief discussion. If peer coaches are responsible for demonstrating and leading the exercises but are not formally trained in the supportive or autonomous principles, this lack of training directly influences the "unknown" or "low" compliance scores for those specific SAAFE domains observed in Table 1. Adding a brief sentence in the discussion section that explicitly connects the limited scope of the peer coach training to these specific fidelity outcomes would provide a more cohesive and comprehensive interpretation of the implementation barriers.

Response:

Thank you for the constructive suggestion. Indeed, we only provided training of SAAFE principles among PE teachers and research assistants who conducted the data collection. We have added this in the discussion in lines 464 to 469, as shown below:

*It should be noted that the peer coaches did not receive training in the SAAFE principles during the training workshop, and we did not conduct on-site observations to evaluate the implementation of the principles in practice among PE teachers or researcher assistants (e.g., encourage mutual support or minimise controlling language). Therefore, the compliance to some of these principles remains unknown or low, which should not be overlooked in future investigations.*

- Finally, while the main text is written in clear and standard English, a meticulous review of the bibliography reveals several formatting inconsistencies and metadata extraction errors indicative of unverified reference management software output. Submitting a manuscript at this advanced stage without a thorough manual proofread of the references does not align with the rigorous publication standards expected by the journal. For instance, Reference 5 displays the journal name in all capital letters and lacks standard page numbers. Reference 37 contains a formatting anomaly in the author name, listing "K BO" instead of the correct format. Reference 35 utilizes inconsistent capitalization for the journal title, and Reference 41 is missing volume and page numbers entirely. A manual, line-by-line proofread of the entire reference list is required to ensure all citations strictly adhere to the formatting guidelines, presenting a polished and professional final manuscript. Implementing these minor adjustments will ensure the manuscript perfectly reflects the high quality of the underlying research.

**Response:**

We greatly appreciate your careful review of the reference list. We have reviewed and made changes accordingly, as listed below.

5.	Hay J, Maximova K, Durksen A, Carson V, Rinaldi RL, Torrance B, et al. Physical activity intensity and cardiometabolic risk in youth. *Archives of Pediatrics & Adolescent Medicine.* 2012;166(11):1022–1029.

15.	Lubans DR, Lonsdale C, Cohen K, Eather N, Beauchamp MR, Morgan PJ, et al. Framework for the design and delivery of organized physical activity sessions for children and adolescents: rationale and description of the ‘SAAFE’ teaching principles. *International Journal of Behavioral Nutrition and Physical Activity*. 2017;14(1):1-11.

19.	Kennedy SG, Leahy AA, Smith JJ, Eather N, Hillman CH, Morgan PJ, et al. Process Evaluation of a School-Based High-Intensity Interval Training Program for Older Adolescents: The Burn 2 Learn Cluster Randomised Controlled Trial. *Children (Basel).* 2020;7(12):299.

20.	Liu Y, Williams CA, Weston KL, Duncombe SL, Malik AA, Barker AR. Validation and calibration for embedding rating of perceived exertion into high-intensity interval exercise in adolescents: a lab-based study. *Pediatric Exercise Science*. 2024;37(1):11-17.

21.	Liu Y, Barker AR, Li M, Gu Y, Williams CA. Validation of polar verity sense for heart rate monitoring during school-based high-intensity interval training in adolescents. *Journal of Sports Sciences.*  2025;43(11):1076-1084.

29.	Lubans DR, Smith JJ, Eather N, Leahy AA, Morgan PJ, Lonsdale C, et al. Time-efficient intervention to improve older adolescents' cardiorespiratory fitness: findings from the 'Burn 2 Learn' cluster randomised controlled trial. *British Journal of Sports Medicine.* 2021;55(13):751–758.

33.	Weston KL, Innerd A, Azevedo LB, Bock S, Batterham AM. Process Evaluation of Project FFAB (Fun Fast Activity Blasts): A Multi-Activity School-Based High-Intensity Interval Training Intervention. *Frontiers in Sports and Active Living*. 2021;3:737900.

36.	Owen KB, Smith J, Lubans DR, Ng JY, Lonsdale C. Self-determined motivation and physical activity in children and adolescents: a systematic review and meta-analysis. *Preventive Medicine*. 2014;67:270-9.

40.	Li M, Liu Y, Gu Y, Sum RKW. Physical activity interventions among children and adolescents in China: a scoping review through an equity lens. *International Journal of Behavioral Nutrition and Physical Activity*. 2025;23(1):9.
