# Supplementary material for: Understanding the “how” and “why”: A mixed methods process evaluation for the PRO-HIIT intervention
Source: PLoS One. 2026 Jun 30;21(6):e0352772. doi: 10.1371/journal.pone.0352772 (PMC13318028; doi:10.1371/journal.pone.0352772)
Supplement: Attachment — Submitted filename: Response to reviewers.docx [file pone.0352772.s005.docx]

6 January 2026

Dear reviewers,

**RE: REVISION, PLOS ONE, PONE-D-25-62980 “Understanding the “how” and “why”: a mixed methods process evaluation for the PRO-HIIT intervention”.**

Thank you very much for your thorough review of our article and the opportunity to address your comments. In this point-by-point response, we have repeated your comments and then provided our response accordingly. All revisions made to the manuscript in response to your feedback have been highlighted in yellow, and the relevant revised text is repeated in this document in *italics*, where appropriate.

We look forward to hearing your thoughts on our revised manuscript.

Kind regards,

The authors

**Reviewer #1:**

**General comments to the authors**

- The manuscript is nicely written, and the topic is valuable and important. However, I have a few major concerns which should be addressed. First, and the most important one, is the definition of the training program studied. Namely, HIIT is rather strictly defined training program which is used with the main purpose of increasing VO2max (Buchheit & Laursen, 2013). The main acute physiological stimulus required to enable adaptation is time spent ≥90% VO2max/HRmax (Buchheit & Laursen, 2013). To enable attainment and maintenance of intensity which would elicit VO2 and HR responses above 90% of their respective maximal values, the exercises used are often cyclic in nature and the intensity is strictly prescribed through velocity (for running) or power (for cycling) defined through some kind of progressive incremental test. While other body weight cyclic exercises (like the ones you used in your study) can lead to high level of intensities, the intensity is hard to prescribe and control (monitor) for. Therefore, these exercises are not used for HIIT as they are usually not able to elicit the appropriate level of intensity nor they can enable maintenance of that intensity for, for example, 5 minutes (which is proposed as the low threshold for acute physiological response to HIIT). Of course, your other references (like #19), point out that you very well know all this stuff. My main suggestion here is, therefore, to “rename” the training program so it is clear from the get-go that body-weight cyclic exercises were used in the training program, and not the actual HIIT. This could be addressed in the title and throughout the manuscript.

**Response:**

Thank you very much for you time in reviewing our manuscript and for your insightful comments. We appreciate you concern regarding the use of term “HIIT” and your kind suggestion to address this concern. We agree HIIT is a form of exercise that strict control and monitor of intensity is required. However, we would like to clarify that your points largely reflect a laboratory-based perspective of HIIT, whereas the present study was conducted in a real-world school setting. In school-based practices, the strict prescription and control of intensity via speed or power output, as well as the use of cyclic modalities such as treadmill running or cycling, are often not feasible due to logistical, pedagogical, and safety constraints. Consequently, body-weight, multi-joint, cyclic activities are commonly adopted in school interventions and have been widely used in previous school-based HIIT research.

In addition, we note that HIIT is not yet universally or unequivocally defined in the literature. While Buchheit and Laursen (2013) emphasise ≥90% VO₂_max_/HR_max_ as a key stimulus, other studies have operationalised HIIT using different criteria. For example, Bond et al. (2017) defined HIIT intensity as ≥70% VO_2peak_, which demarcates the upper boundary of the moderate-intensity domain and represents an intensity greater than the lactate threshold. This variability indicates that the definition of HIIT remains context-dependent, particularly when transitioning from laboratory to applied settings such as schools. For example, Duncombe et al. (2024) utilised 80% HR_max_ and Kennedy et al. (2020) adopted 85% HR_max_, respectively as thresholds for HIIT in school settings.

Moreover, and most importantly, this manuscript is a process evaluation of a school-based HIIT intervention, examining whether the intervention was delivered as intended in relation to our predefined protocol. The primary aim was not to evaluate physiological adaptations (e.g., VO2max improvements), but to assess process evaluation components such as fidelity, dose delivered and received, and response from participants in a school environment. From this perspective, an extended debate regarding the optimal physiological definition of HIIT is largely beyond the scope of the present paper.

We acknowledge the reviewer’s concern regarding terminology and have clarified throughout the manuscript that the intervention consisted of body-weight-based high-intensity interval activities implemented in a school setting, rather than laboratory-prescribed HIIT aimed specifically at maximising VO_2max_. We believe this clarification addresses potential ambiguity while retaining terminology that is consistent with previous school-based HIIT literature.

- Secondly, it is unclear whether you have and how you assessed HRmax? The 20 m shuttle run test was mentioned in the Discussion section; however, it was not mentioned in the Methods. So, did you perform the 20mSRT or any other maximal incremental aerobic test to establish HRmax?

**Response:**

We would like to clarify that HR_max_ was not directly assessed using a maximal incremental test (e.g., 20m-SRT), given the time constraints and limited resources. Instead, we used age-predicted HRmax by applying the widely used equation HRmax = 220 – age. We have clarified this in the methods in lines 184-185.

*The HIIT intensity threshold was set at HR ≥ 80% HR_max_ (with individual HR_max_ calculated using* *220-age).*

- Thirdly, some of the instruments used to assess, for example, enjoyment, were not supported by references. So, what instruments were used? Were they validated? References to support their validity and reliability. Overall, there are number of variables presented in the manuscript for which we do not know how they were measured.

**Response:**

We would like to clarify that the instruments used to assess enjoyment and continue use of HIIT were adopted from previously published studies in this field (e.g., Duncombe et al. 2024 and Costigan et al. 2015), and the relevant references have now been cited in the manuscript. We have revised the manuscript to clearly specify the instruments used:

*Response was the students and teachers’ reaction and attitude towards the PRO-HIIT intervention. It was assessed qualitatively via student and teacher’s focus groups as well as quantitatively on a 2-item 5-point Likert scale, which has been used in previous school-based HIIT interventions for assessing satisfaction (1, 2). The scale started with the prompt: “I enjoyed/liked the HIIT workouts” and “I will continue to perform/use the HIIT workouts”, with the score ranged between 1 = strongly disagree and 5 = strongly agree.*

- Finally, I think that the manuscript can be defined as professional paper at best, as no quantitative or appropriate qualitative analysis was performed. It was probably due to the low number of participants included in the focus groups and interviews. Unfortunately, this is the main drawback of the study. Even though you conducted a large-scale intervention which engaged large number of participants, the overall data collected was just not enough for making and firm conclusions. We did not learn much from the study.

**Response:**

We acknowledge your concern regarding the depth of the qualitative data and agree that the number of participants included in the focus groups and interviews may not have been sufficient to reach data saturation. This limitation arose due to practical constraints inherent to real-world school-based research – our post-intervention data collection coincided with the final two weeks of the semester, a period dominated by examinations and academic commitments, during which participation in focus groups was deprioritised by both students and teachers. We have clarified this issue in the **Strength and limitations** section upon our first submission.

However, despite the lower-than-expected qualitative sample, the data were analysed based on valid and robust guidelines (Braun and Clarke, 2006), and we believe these findings still offer original and meaningful insights into intervention delivery, acceptability, and areas for refinement. These insights are particularly relevant for informing future implementation and potential scale-up of similar school-based interventions, which is a key objective of process evaluation research. Similar small numbers of student focus groups and teacher interviews for process evaluation studies in school settings also exist in the literature, such as Taylor et al. 2018 (student: n=16, teacher: n=3) and Harris et al. 2019 (student: n=9, teacher: n=4).

Regarding the comment on the absence of quantitative analysis, we have to clarify the data related to HR, RPE, enjoyment and continue to use of HIIT, does delivered and received are calculated quantitatively.

**Specific comments to the authors**

- Line 129: Something is wrong here.

**Response:**

We have clarified the sentence with the following:

*Three hundred and sixty-nine Year 7 students (aged 12–13 years) from eight different classes were included in the intervention, all of whom provided written assent and parental consent.*

- 2. Line 233: Remove “is” or “was” from the sentence.

**Response:**

Sorry for the type error. We have removed “is” from the manuscript.

- 3. Line 303: “May be too long” should be here.

**Response:**

Thank you very much for correcting us. We have changed this to “May be too long”.

- 4. Line 425-427: I would disagree that there is no consensus reached about the main acute parameter which would serve as the HIIT target. Namely, time spent above 90% HRmax is currently used as proxy measure to achieve optimal cardiorespiratory stimulus during HIIT (Buchheit & Laursen, 2013).

**Response:**

We acknowledge that time spent ≥90% HRmax has been widely used as a proxy for achieving a strong cardiorespiratory stimulus in laboratory-based HIIT research, as outlined by Buchheit and Laursen (2013). However, we respectfully disagree that this represents a universally accepted consensus of HIIT parameter.

The framework proposed by Buchheit and Laursen (2013) is largely derived from laboratory-based studies employing tightly controlled exercise modalities (e.g., treadmill running or cycling) with precisely prescribed intensities based on findings mostly from adult or athlete populations. While highly influential, this perspective may not be fully transferable to applied, real-world settings, such as schools, where strict control of workload, modality, and sustained intensity is often not feasible. It is also not feasible to retain children and adolescent participants under such high exercise intensity.

Additionally, the Buchheit and Laursen (2013) paper, although seminal, is now over a decade old, and more recent literature has highlighted the heterogeneity in HIIT intensity prescription and monitoring, particularly in youth and field-based interventions (see studies by Viana et al. 2018, Cadenas-Sanchez et al. 2024 and Bond et al. 2017). As Cadenas-Sanchez et al. (2024) commented on Buchheit’s study: “However, the acceptance of the HIIT concept and its dimensions were not universally accepted, leading to diverse definitions in the literature. Such heterogeneity has resulted in multiple exercise protocols and exertion criteria and descriptions. Moreover, these dimensions did not consider the physiological nuances of non-athlete populations and their interactions with health outcomes. (p 401)”

These points suggest that while ≥90% HRmax remains a commonly used laboratory proxy, it should not be interpreted as a definitive or universally agreed-upon criterion across all contexts. However, as stated above, these debates should not be taken place in the present study, in which the main purpose is to understand if the implementation process of an intervention. Therefore, we have removed this argument in the discussion:

*While the intensity achieved in our study might be slightly lower than expected (i.e., ≥80% HR_max_), it deemed to be able to bring meaningful health benefits to children and adolescents (3, 4). ~~In addition, it is pertinent to mention that no consensus has been reached to prescribe HIIT intensity using HR (5).~~*

- 5. One of the conclusions was that dose fell slightly below the minimum. How did you select the minimum (i.e., 5 times/week)? Why would you think that this is the minimum? Reference?

**Response:**

We set 30 HIIT sessions as a minimum dose we expected to deliver in our protocol paper. This number came from both the Chinese school context (e.g., holidays and weather) as well as from previous school-based HIIT interventions (i.e., Kennedy et al 2020). We have clarified this in the **Materials and Methods** of the manuscript (lines 172-175):

*Of note, a minimum of 30 sessions was expected to be delivered over the 12-week period, taking into account the Chinese context (e.g., holidays and weather) as well as informed by a previous intervention (6).*

- 6. Results section should be shortened drastically as no firm conclusions can be made based on all these individual responses presented.

**Response:**

Thank you for the comments. As noted above, although we did not achieve the stratified number of participants for the focus groups, the results, discussion, and conclusions were not derived from individual responses, but from thematic analyses and summary of findings from two focus groups (nine participants in total) and the interview of two PE teachers. For example, all participants in the focus group and teachers we interviewed consistently acknowledged the value of peer coaches, which was therefore identified as a key facilitator for intervention delivery and recommended for future research. Individual quotations presented in the manuscript, such as “*I think they (peer coaches) did a very good job. I mean they could perform those exercises very professionally and know exactly what the next move was*” (Student focus group 2, student B), were selected as illustrative examples of shared perspectives, rather than as standalone individual viewpoints. This approach is commonly used in process evaluation of school-based interventions. Therefore, we wish to keep these contents as they constitute important parts of the present paper. We hope that our rationale is clear and appreciate your understanding.
**Relevant references:**

Bond B, Weston KL, Williams CA, Barker AR. Perspectives on high-intensity interval exercise for health promotion in children and adolescents. Open Access Journal of Sports Medicine. 2017 Nov 27:243-65.

Viana RB, de Lira CA, Naves JP, Coswig VS, Del Vecchio FB, Ramirez-Campillo R, Vieira CA, Gentil P. Can we draw general conclusions from interval training studies?. Sports Medicine. 2018 Sep;48(9):2001-9.

Cadenas-Sanchez C, Fernandez-Rodriguez R, Martinez-Vizcaino V, de los Reyes Gonzalez N, Lavie CJ, Galan-Mercant A, Jimenez-Pavon D. A systematic review and cluster analysis approach of 103 studies of high-intensity interval training on cardiorespiratory fitness. European Journal of Preventive Cardiology. 2024 Mar;31(4):400-11.

Duncombe SL, Barker AR, Price L, Walker JL, Koep JL, Woodforde J, et al. Was it a HIIT? A process evaluation of a school-based high-intensity interval training intervention. International Journal of Behavioral Nutrition and Physical Activity. 2024;21(1):49.

Kennedy SG, Leahy AA, Smith JJ, Eather N, Hillman CH, Morgan PJ, et al. Process evaluation of a school-based high-intensity interval training program for older adolescents: the burn 2 learn cluster randomised controlled trial. Children. 2020;7(12):299.

**Reviewer #2:**

Dear Authors,

Thank you for submitting this manuscript that presents a mixed methods process evaluation of the PRO-HIIT intervention within a Chinese secondary school setting. I commend the authors for moving beyond simple efficacy testing to explore the "black box" of intervention implementation. The application of the Medical Research Council (MRC) guidance provides a robust theoretical framework for this study, and the honesty regarding implementation barriers, such as weather and conflicting school schedules—adds significant ecological validity to the work. The findings regarding the trade-off between intervention fidelity and student autonomy are particularly insightful for the field of pediatric exercise science. However, to strengthen the manuscript and ensure it meets the highest standards of reporting, I have identified specific areas requiring clarification and refinement.

**Response:**

Thank you very much for your positive and thoughtful assessment of our manuscript. We greatly appreciate your recognition of the often-missed part of process evaluation and the adoption of guidelines for reporting it in school-based PA interventions. In response to the specific areas identified for clarification and refinement, we have carefully revised the manuscript accordingly and addressed each comment in detail below.

- In the Methods section, regarding the qualitative component, you note on lines 214–218 that two student focus groups (n=9 total) and one teacher focus group (n=2) were conducted. While mixed methods research often relies on smaller qualitative samples, you must explicitly address whether data saturation was achieved with this sample size. With only two teachers interviewed, it is crucial to justify how this represents the broader experience of the staff involved, or acknowledging this as a limitation in capturing the full breadth of the "teacher experience."

**Response:**

Thank you for your insightful comments regarding the sample size for focus groups. We agree that data saturation is an important consideration in qualitative research. With regard to the teacher component, all physical education teachers involved in the delivery of the intervention (n = 3) were invited to participate in the qualitative evaluation. Two teachers completed the interview, while one teacher withdrew during the interview due to competing responsibilities. As such, the participating teachers represent the majority of the intervention delivery staff, and their perspectives provide insight into key implementation issues encountered during the intervention. Nevertheless, we acknowledge that the inclusion of only two teachers may not fully capture the breadth of teacher experiences, and this has now been explicitly acknowledged as a limitation in the manuscript under the **Strength and limitations** section:

*Furthermore, only nine participants and two PE teachers were involved in the focus groups, which is lower than the recommended sample sizes for qualitative research and may have limited the breadth and depth of perspectives captured on the topics explored (7).*

- Furthermore, on line 148, you mention peer coaches were trained to lead sessions. Given that "peer coaches" emerged as a facilitator in your results, the Methods section requires more detail on the specific content and duration of their training. Did the peer coaches receive specific instruction on the SAAFE principles? Providing this detail is essential for replication.

**Response:**

Thank you for the instructive suggestions here. The main jobs for peer coaches in the HIIT session delivery is to demonstrate the HIIT moves so that the other participants can follow. As we used multiple different moves for each HIIT session, this demonstration would have set as examples to guide and motivate participants. However, they are not responsible for communicating, organising or keeping the sessions in order, given they may feel incompetent to do so and the PE teachers normally present and ready for this job. Therefore, we did not include SAAFE principles in the training session for peer coaches. Instead, the peer coaches’ training included how to perform the HIIT moves, how to help PE teachers to take the attendance or collect RPE data and so on. A detailed table for the time and content of the training workshop is provided in our protocol paper. Therefore, we have referred the reader to the protocol paper for more details:
*The peer coaches were trained to demonstrate and lead the HIIT sessions in front of the class, as well as help with other tasks, such as taking attendance, which varied at the discretion of the PE teachers. More details regarding the training workshops can be found in our protocol paper (8).*

- Regarding the data analysis and results, there is a minor typographical error on line 233 stating "The coding is was initially deductive," which requires correction.

**Response:**

Thank you for the correction. We have removed “is” from the text.

- More substantially, in the Fidelity section (lines 262–267), the presentation of heart rate (HR) data requires precision. You state that 18% of HR measures met the 80% HRmax threshold, yet the mean peak HR was 85% HRmax. It is important to clarify if the "18%" refers to the total time spent above the threshold or the percentage of individual data points collected that were above the threshold. If students are reaching a peak of 85% but the average is 71%, this suggests the interval peaks are high but recovery is substantial. Please ensure the distinction between "time spent at intensity" versus "peak intensity achieved" is explicit, as this speaks directly to the physiological stimulus delivered.

**Response:**

Thank you very much for this insightful comment. We used two criteria, individual mean HR and individual peak HR, to evaluate if participants achieved prescribed intensity threshold – 80%HRmax. This is because these two criteria are commonly used in the literature of school-based HIIT interventions. We appreciate your comments on clarification of these points, and we have changed the manuscript to:

*In total, HR measures from 127 individuals were observed across 13 HIIT sessions and RPE measures were collected from 225 individuals across 5 HIIT sessions. The average HR was 146 ± 17 beats per minute, corresponding to 71% HR_max_. Among these individual measures, 18% (n = 23) of them meeting the 80% HR_max_ threshold. The HR peak achieved by these individuals were also observed, with a mean of 175 ± 17 beats per minute (85% HR_max_), and 79% of them meeting the HIIT threshold.*

- In the Discussion section, the interpretation of the results is generally sound and well-contextualized. However, the discussion regarding the "Repetition of HIIT workouts" (Line 372) as a barrier could be deepened. You mention that students found it monotonous, yet retention was high. It would be valuable to discuss this paradox further. Does the social element or the peer-coaching aspect mitigate the boredom of repetitive exercises?

**Response:**

Thank you for helping us to dig deeper into this aspect. As discussed later in the manuscript, the school involved is among the best in the local city and the students are very well-behaved. This means that they were unlikely to drop out if most students are doing it, instead, reduced engagement or motivation was more likely to be expressed if they feel bored. We have added a sentence to discuss this in the manuscript in lines 516-519:

*Although the PRO-HIIT intervention offered more than ten different workouts,* *focus group findings suggested that this variety was insufficient across the twelve-week programme. Consequently, student engagement or motivation may have been somewhat reduced as the providing of variety is key to the enjoyment and motivation for engaging in HIIT (9). Interestingly, this finding appears to contrast with the high retention rate, which is likely influenced by social factors, such as social conformity or the desire to perform well in front of teachers.*

- Additionally, on line 424, you suggest the intensity might be "slightly lower than expected." I would argue that for a school-based, peer-led intervention, a mean HR of 71% HRmax is actually quite successful. You might frame this more positively as a demonstration of feasibility in a non-laboratory setting, rather than a failure to meet strict laboratory HIIT criteria which are often unrealistic in schools.

**Response:**

Thank you for the thoughtful suggestion. Perhaps "slightly lower than prescribed" is more appropriate in this context and we have revised the manuscript accordingly. We have also added an additional sentence to further clarify and refine this point (lines 431-433):

*While the intensity achieved in our study might be slightly lower than prescribed (i.e., ≥80% HR_max_), it should be considered a meaningful achievement for a school-based, peer-led intervention, which is likely sufficient to confer meaningful health benefits for children and adolescents (3, 4).*

- Finally, regarding the "Unintended Consequences" (Line 196 and 363), you list the school-wide sports event disrupting dosage as an unintended consequence. In the context of process evaluation, this is perhaps better categorized strictly as a contextual barrier or implementation failure rather than an "unintended consequence" of the intervention itself (which usually refers to adverse events or unexpected positive side effects). Reconsidering this categorization might align better with the MRC framework's intent. Addressing these points will clarify the contribution of this work to the field of implementation science in youth physical activity.

**Response:**

Thank you for this helpful clarification. We agree that the disruption caused by the school-wide sports event is more appropriately conceptualized as a contextual barrier, rather than an unintended consequence of the intervention itself. We, therefore, have moved this to the **Barriers** section:

***Barriers***

***Competing priorities.*** *From the fifth intervention week onwards, HIIT sessions in PA lessons were disrupted owing to a school-wide sports event, contributing to the lower dose delivered than planned.* ***Severe weather.*** *Both hot weather and rain threatened the implementation of the PRO-HIIT intervention. On hot days, session quality declined, while rainy days led to cancellation of PE lessons.*
