# Supplementary material for: Understanding the “how” and “why”: A mixed methods process evaluation for the PRO-HIIT intervention
Source: PLoS One. 2026 Jun 30;21(6):e0352772. doi: 10.1371/journal.pone.0352772 (PMC13318028; doi:10.1371/journal.pone.0352772)
Supplement: Attachment — Submitted filename: Response to Reviewers_v3.docx [file pone.0352772.s007.docx]

9 June 2026

**RE: REVISION, PLOS ONE, PONE-D-25-62980 “Understanding the “how” and “why”: a mixed methods process evaluation for the PRO-HIIT intervention”.**

To the Editor and Dr. Alston Choong [named reviewer],

We sincerely thank you for your positive and thoughtful evaluation of our manuscript. We greatly appreciate the recognition of the value of the process evaluation, the application of the Medical Research Council (MRC) framework, and the rationale underpinning the design and implementation of the PRO-HIIT intervention.

We appreciate your constructive recommendations regarding alignment with the Good Reporting of A Mixed Methods Study (GRAMMS) framework and the Consolidated Criteria for Reporting Qualitative Research (COREQ) guidelines. In response, we have revised the manuscript to further strengthen methodological transparency and reporting quality.

We have repeated your comments below only for those revisions are required and provided a response accordingly with changes to the manuscript were highlighted in yellow.

We hope we have satisfied your queries satisfactorily and we look forward to a decision about the revised manuscript.

Kind regards,

The authors

1. In the Methods section, the detailed mapping of the process evaluation measures to the MRC framework is a significant strength. However, a minor methodological flaw remains regarding the explicit classification of the mixed methods approach. While it is correctly stated that both quantitative and qualitative data were collected, the manuscript lacks a formal declaration of the specific mixed methods design typology employed (for instance, a convergent parallel design, an explanatory sequential design, or an exploratory sequential design). To align with the Good Reporting of A Mixed Methods Study (GRAMMS) framework, it is recommended that the authors explicitly define their mixed methods design in the Data Analysis section. A brief explanation of how and at what stage the quantitative and qualitative data were integrated to formulate the conclusions should be provided.

**Response:**

Thank you for this helpful suggestion and for highlighting the importance of explicitly reporting the mixed methods design in accordance with the GRAMMS framework. In response, we have revised the Data Analysis section to specify that the study employed a **convergent parallel mixed methods design**, whereby quantitative and qualitative data were collected concurrently, analysed independently, and integrated during the interpretation stage. We have also added a description of the integration process, clarifying that findings from both datasets were triangulated across the Medical Research Council (MRC) process evaluation domains and jointly interpreted to identify areas of convergence, complementarity, and divergence. These integrated findings informed the overall conclusions regarding the implementation, acceptability, and contextual influences of the PRO-HIIT intervention.

**The Data Analysis section of the manuscript has been changed to:**

*The current study employed a convergent parallel mixed methods design. Quantitative data were collected for reach, recruitment and retention, dose delivered, fidelity, dose received and response. Data were analysed using IBM SPSS Statistics for Windows (SPSS 28.0; IBM Corporation, Armonk, NY, USA) unless specified. Qualitative data were collected for adaptation, response, dose received, unintended consequence, barriers and facilitators, and contamination. The focus group recordings were transcribed verbatim in Chinese and were translated to English by the first author. A second author (MHL) checked the transcribed data for completeness and accuracy. Transcripts were analysed using thematic analysis following a six-phase guidance (i.e., adaptation, response, barriers, facilitators, and contamination). The coding was initially deductive based on the MRC guidance, followed by an inductive process directed by the content of the data (25). To maximise consistency and completeness, two authors (YL and SLD) independently conducted the thematic analysis using NVivo software (Version 15) and discussed their findings until consensus was reached. Integration of quantitative and qualitative findings occurred during the interpretation stage. Areas of convergence, complementarity, and divergence between the quantitative and qualitative findings were jointly interpreted to inform the overall conclusions of the process evaluation.*

2. Furthermore, while the qualitative methodology involving focus groups and thematic analysis (using the Braun and Clarke approach) is sound, there is no explicit mention of adherence to the Consolidated Criteria for Reporting Qualitative Research (COREQ), which is the standard EQUATOR Network guideline for qualitative studies. As an actionable recommendation, the authors should insert a brief statement confirming adherence to the COREQ guidelines. In doing so, the concept of data saturation within the qualitative data subsection must be briefly addressed. Given the small qualitative sample size (nine students and two teachers), it is important to explicitly state whether thematic saturation was reached or if the findings represent an exploratory thematic overview rather than an exhaustively saturated dataset. This transparency will protect the manuscript from future methodological critiques regarding qualitative sample size adequacy.

**Response:**

We thank the reviewer for this valuable suggestion regarding qualitative reporting transparency. We agree that the COREQ checklist provides important guidance for reporting qualitative research. However, the qualitative component of the present study was embedded within a broader mixed methods process evaluation and was not prospectively designed or reported using the COREQ framework. Therefore, we believe it would not be appropriate to retrospectively claim adherence to COREQ when the study was not originally conducted and documented according to its reporting criteria.

Nevertheless, we appreciate the reviewer's concern regarding qualitative sample adequacy and thematic saturation. In response, we have revised the Strengths and Limitations section to explicitly acknowledge that the qualitative sample comprised only nine students and two teachers and that exhaustive thematic saturation was not the primary objective of this process evaluation. We now clarify that the qualitative findings should be interpreted as an exploratory thematic overview intended to complement the quantitative process evaluation and provide contextual insights into implementation experiences, barriers, facilitators, and participant responses.

**The Strength and Limitations section has been changed to:**

*The current study utilised a convergent parallel mixed methods approach, guided by the MRC framework to give a complete picture of the process evaluation. The integration of quantitative and qualitative data enabled triangulation across process evaluation domains and reduced the likelihood of incomplete reporting or biased interpretation. However, several limitations should be acknowledged. First, the students and teachers were not blinded to the intervention groups and the session intensity were only measured in a sub-sample of participants and in PE lessons only. This could potentially contaminate or obscure our research findings. Second, several process evaluation outcomes were assessed using subjective measures (e.g., session quality scores), which may have been susceptible to social desirability bias. Third, the qualitative sample comprised only nine students across two focus groups and two PE teachers who participated in individual interviews. Although these data provided valuable insights into participants’ experiences and perceptions of the intervention, the sample size may have limited the breadth and diversity of perspectives captured. The qualitative component was designed to complement the quantitative process evaluation rather than to achieve exhaustive thematic saturation (44). Therefore, the qualitative findings should be interpreted as an exploratory thematic overview of key implementation experiences, barriers, facilitators, and perceived impacts within this context**.*

3. In conclusion, this is a highly commendable manuscript that makes a significant contribution to the literature on physical activity interventions in adolescents. The recommendation for a minor revision is strictly to allow the authors to explicitly align their methodology section with the GRAMMS and COREQ reporting guidelines, specifically by naming their mixed methods design typology and clarifying qualitative thematic saturation, thereby ensuring full compliance with the EQUATOR Network's international reporting standards.

**Response:**

Thank you for your positive appraisal of our manuscript and for recognising its contribution to the literature on school-based physical activity interventions. We appreciate the constructive recommendations regarding reporting transparency and methodological clarity. In response, we have revised the manuscript to explicitly identify the study as a **convergent parallel mixed methods design**. We have also added an extra sentence to acknowledge the exploratory nature of the qualitative findings and to highlight future research directions.

**The Conclusion section has been changed to:**

*The present study provides valuable insights into the “how” and “why” the PRO-HIIT intervention works or not within a Chinese secondary school setting, utilising a convergent parallel mixed methods process evaluation. Overall, the PRO-HIIT intervention demonstrated favourable reach, retention, and dose received as indicated by the positive responses from both teachers and students. However, the dose delivered fell slightly below the predefined threshold mainly due to uncontrollable competing priorities, and implementation fidelity was only partially achieved as intended. Our study demonstrated the potential for incorporating HIIT in existing PA curriculum time to engage Chinese children and adolescents in vigorous PA. As a reflection, the implementation of the PRO-HIIT intervention may have come at the expense of students’ autonomy. Future studies should aim to better balance implementation fidelity and autonomy support and seek to foster PA motivation through intrinsic means. Given the exploratory nature of the qualitative component, further research involving larger and more diverse qualitative samples is warranted to confirm and extend the implementation insights identified in the present study.*
