# Supplementary material for: Understanding the “how” and “why”: A mixed methods process evaluation for the PRO-HIIT intervention
Source: PLoS One. 2026 Jun 30;21(6):e0352772. doi: 10.1371/journal.pone.0352772 (PMC13318028; doi:10.1371/journal.pone.0352772)
Supplement: Peer review history

# Peer review: Decision Letter 0

**DOI:** 10.1371/journal.pone.0352772.r001

**Authors:** Esedullah Akaras

29 Dec 2025

PONE-D-25-62980

Understanding the “how” and “why”: a mixed methods process evaluation for the PRO-HIIT intervention

PLOS One

Dear Dr. Liu,

Thank you for submitting your manuscript to PLOS ONE. After careful consideration, we feel that it has merit but does not fully meet PLOS ONE’s publication criteria as it currently stands. Therefore, we invite you to submit a revised version of the manuscript that addresses the points raised during the review process.

Please submit your revised manuscript by Feb 12 2026 11:59PM. If you will need more time than this to complete your revisions, please reply to this message or contact the journal office at plosone@plos.org. When you're ready to submit your revision, log on to https://www.editorialmanager.com/pone/ and select the 'Submissions Needing Revision' folder to locate your manuscript file.

Please include the following items when submitting your revised manuscript:

- A letter that responds to each point raised by the academic editor and reviewer(s). You should upload this letter as a separate file labeled 'Response to Reviewers'.
- A marked-up copy of your manuscript that highlights changes made to the original version. You should upload this as a separate file labeled 'Revised Manuscript with Track Changes'.
- An unmarked version of your revised paper without tracked changes. You should upload this as a separate file labeled 'Manuscript'.

If you would like to make changes to your financial disclosure, please include your updated statement in your cover letter. Guidelines for resubmitting your figure files are available below the reviewer comments at the end of this letter.

If applicable, we recommend that you deposit your laboratory protocols in protocols.io to enhance the reproducibility of your results. Protocols.io assigns your protocol its own identifier (DOI) so that it can be cited independently in the future. For instructions see: https://journals.plos.org/plosone/s/submission-guidelines#loc-laboratory-protocols. Additionally, PLOS ONE offers an option for publishing peer-reviewed Lab Protocol articles, which describe protocols hosted on protocols.io. Read more information on sharing protocols at https://plos.org/protocols?utm_medium=editorial-email&utm_source=authorletters&utm_campaign=protocols.

We look forward to receiving your revised manuscript.

Kind regards,

Esedullah Akaras

Academic Editor

PLOS One

**Journal Requirements:**

1. When submitting your revision, we need you to address these additional requirements.

Please ensure that your manuscript meets PLOS ONE's style requirements, including those for file naming. The PLOS ONE style templates can be found at

https://journals.plos.org/plosone/s/file?id=wjVg/PLOSOne_formatting_sample_main_body.pdf and

https://journals.plos.org/plosone/s/file?id=ba62/PLOSOne_formatting_sample_title_authors_affiliations.pdf

2. Please upload a new copy of Figures 1 – 5 as the detail is not clear. Please follow the link for more information:  https://journals.plos.org/plosone/s/figures

3. Please include captions for your Supporting Information files at the end of your manuscript, and update any in-text citations to match accordingly. Please see our Supporting Information guidelines for more information: http://journals.plos.org/plosone/s/supporting-information.

4. We note that this data set consists of interview transcripts. Can you please confirm that all participants gave consent for interview transcript to be published?

If they DID provide consent for these transcripts to be published, please also confirm that the transcripts do not contain any potentially identifying information (or let us know if the participants consented to having their personal details published and made publicly available). We consider the following details to be identifying information:

- Names, nicknames, and initials

- Age more specific than round numbers

- GPS coordinates, physical addresses, IP addresses, email addresses

- Information in small sample sizes (e.g. 40 students from X class in X year at X university)

- Specific dates (e.g. visit dates, interview dates)

- ID numbers

Or, if the participants DID NOT provide consent for these transcripts to be published:

- Provide a de-identified version of the data or excerpts of interview responses

- Provide information regarding how these transcripts can be accessed by researchers who meet the criteria for access to confidential data, including:

a) the grounds for restriction

b) the name of the ethics committee, Institutional Review Board, or third-party organization that is imposing sharing restrictions on the data

c) a non-author, institutional point of contact that is able to field data access queries, in the interest of maintaining long-term data accessibility.

d) Any relevant data set names, URLs, DOIs, etc. that an independent researcher would need in order to request your minimal data set.

For further information on sharing data that contains sensitive participant information, please see: https://journals.plos.org/plosone/s/data-availability#loc-human-research-participant-data-and-other-sensitive-data

If there are ethical, legal, or third-party restrictions upon your dataset, you must provide all of the following details (https://journals.plos.org/plosone/s/data-availability#loc-acceptable-data-access-restrictions):

a) A complete description of the dataset

b) The nature of the restrictions upon the data (ethical, legal, or owned by a third party) and the reasoning behind them

c) The full name of the body imposing the restrictions upon your dataset (ethics committee, institution, data access committee, etc)

d) If the data are owned by a third party, confirmation of whether the authors received any special privileges in accessing the data that other researchers would not have

e) Direct, non-author contact information (preferably email) for the body imposing the restrictions upon the data, to which data access requests can be sent

5. If the reviewer comments include a recommendation to cite specific previously published works, please review and evaluate these publications to determine whether they are relevant and should be cited. There is no requirement to cite these works unless the editor has indicated otherwise.

[Note: HTML markup is below. Please do not edit.]

Reviewers' comments:

Reviewer's Responses to Questions

**Comments to the Author**

1. Is the manuscript technically sound, and do the data support the conclusions?

The manuscript must describe a technically sound piece of scientific research with data that supports the conclusions. Experiments must have been conducted rigorously, with appropriate controls, replication, and sample sizes. The conclusions must be drawn appropriately based on the data presented.

Reviewer #1: Partly

Reviewer #2: Yes

**********

2. Has the statistical analysis been performed appropriately and rigorously?

Reviewer #1: N/A

Reviewer #2: Yes

**********

3. Have the authors made all data underlying the findings in their manuscript fully available?

The PLOS Data policy requires authors to make all data underlying the findings described in their manuscript fully available without restriction, with rare exception (please refer to the Data Availability Statement in the manuscript PDF file). The data should be provided as part of the manuscript or its supporting information, or deposited to a public repository. For example, in addition to summary statistics, the data points behind means, medians and variance measures should be available. If there are restrictions on publicly sharing data—e.g. participant privacy or use of data from a third party—those must be specified.

Reviewer #1: Yes

Reviewer #2: Yes

**********

4. Is the manuscript presented in an intelligible fashion and written in standard English?

PLOS ONE does not copyedit accepted manuscripts, so the language in submitted articles must be clear, correct, and unambiguous. Any typographical or grammatical errors should be corrected at revision, so please note any specific errors here.

Reviewer #1: Yes

Reviewer #2: Yes

**********

5. Review Comments to the Author

Please use the space provided to explain your answers to the questions above. You may also include additional comments for the author, including concerns about dual publication, research ethics, or publication ethics. (Please upload your review as an attachment if it exceeds 20,000 characters)

**Reviewer #1:**  General comments to the authors

The manuscript is nicely written, and the topic is valuable and important. However, I have a few major concerns which should be addressed.

First, and the most important one, is the definition of the training program studied. Namely, HIIT is rather strictly defined training program which is used with the main purpose of increasing VO2max (Buchheit & Laursen, 2013). The main acute physiological stimulus required to enable adaptation is time spent ≥90% VO2max/HRmax (Buchheit & Laursen, 2013). To enable attainment and maintenance of intensity which would elicit VO2 and HR responses above 90% of their respective maximal values, the exercises used are often cyclic in nature and the intensity is strictly prescribed through velocity (for running) or power (for cycling) defined through some kind of progressive incremental test. While other body weight cyclic exercises (like the ones you used in your study) can lead to high level of intensities, the intensity is hard to prescribe and control (monitor) for. Therefore, these exercises are not used for HIIT as they are usually not able to elicit the appropriate level of intensity nor they can enable maintenance of that intensity for, for example, 5 minutes (which is proposed as the low threshold for acute physiological response to HIIT). Of course, your other references (like #19), point out that you very well know all this stuff. My main suggestion here is, therefore, to “rename” the training program so it is clear from the get-go that body-weight cyclic exercises were used in the training program, and not the actual HIIT. This could be addressed in the title and throughout the manuscript.

Secondly, it is unclear whether you have and how you assessed HRmax? The 20 m shuttle run test was mentioned in the Discussion section; however, it was not mentioned in the Methods. So, did you perform the 20mSRT or any other maximal incremental aerobic test to establish HRmax?

Thirdly, some of the instruments used to assess, for example, enjoyment, were not supported by references. So, what instruments were used? Were they validated? References to support their validity and reliability. Overall, there are number of variables presented in the manuscript for which we do not know how they were measured.

Finally, I think that the manuscript can be defined as professional paper at best, as no quantitative or appropriate qualitative analysis was performed. It was probably due to the low number of participants included in the focus groups and interviews. Unfortunately, this is the main drawback of the study. Even though you conducted a large-scale intervention which engaged large number of participants, the overall data collected was just not enough for making and firm conclusions. We did not learn much from the study.

Specific comments to the authors

1. Line 129: Something is wrong here.

2. Line 233: Remove “is” or “was” from the sentence.

3. Line 303: “May be too long” should be here.

4. Line 425-427: I would disagree that there is no consensus reached about the main acute parameter which would serve as the HIIT target. Namely, time spent above 90% HRmax is currently used as proxy measure to achieve optimal cardiorespiratory stimulus during HIIT (Buchheit & Laursen, 2013).

5. One of the conclusions was that dose fell slightly below the minimum. How did you select the minimum (i.e., 5 times/week)? Why would you think that this is the minimum? Reference?

6. Results section should be shortened drastically as no firm conclusions can be made based on all these individual responses presented.

Buchheit, M. & Laursen, P.B. (2013). High-intensity interval training, solutions to the programming puzzle. Part 1: Cardiopulmonary emphasis. Sports Medicine, 43(5), 313-338.

Buchheit, M. & Laursen, P.B. (2013). High-intensity interval training, solutions to the programming puzzle. Part 2: Anaerobic energy, neuromuscular load and practical applications. Sports Medicine, 43(10), 927-954.

**Reviewer #2:**  Dear Authors,

Thank you for submitting this manuscript that presents a mixed methods process evaluation of the PRO-HIIT intervention within a Chinese secondary school setting. I commend the authors for moving beyond simple efficacy testing to explore the "black box" of intervention implementation. The application of the Medical Research Council (MRC) guidance provides a robust theoretical framework for this study, and the honesty regarding implementation barriers, such as weather and conflicting school schedules—adds significant ecological validity to the work. The findings regarding the trade-off between intervention fidelity and student autonomy are particularly insightful for the field of pediatric exercise science. However, to strengthen the manuscript and ensure it meets the highest standards of reporting, I have identified specific areas requiring clarification and refinement.

In the Methods section, regarding the qualitative component, you note on lines 214–218 that two student focus groups (n=9 total) and one teacher focus group (n=2) were conducted. While mixed methods research often relies on smaller qualitative samples, you must explicitly address whether data saturation was achieved with this sample size. With only two teachers interviewed, it is crucial to justify how this represents the broader experience of the staff involved, or acknowledging this as a limitation in capturing the full breadth of the "teacher experience." Furthermore, on line 148, you mention peer coaches were trained to lead sessions. Given that "peer coaches" emerged as a facilitator in your results, the Methods section requires more detail on the specific content and duration of their training. Did the peer coaches receive specific instruction on the SAAFE principles? Providing this detail is essential for replication.

Regarding the data analysis and results, there is a minor typographical error on line 233 stating "The coding is was initially deductive," which requires correction. More substantially, in the Fidelity section (lines 262–267), the presentation of heart rate (HR) data requires precision. You state that 18% of HR measures met the 80% HRmax threshold, yet the mean peak HR was 85% HRmax. It is important to clarify if the "18%" refers to the total time spent above the threshold or the percentage of individual data points collected that were above the threshold. If students are reaching a peak of 85% but the average is 71%, this suggests the interval peaks are high but recovery is substantial. Please ensure the distinction between "time spent at intensity" versus "peak intensity achieved" is explicit, as this speaks directly to the physiological stimulus delivered.

In the Discussion section, the interpretation of the results is generally sound and well-contextualized. However, the discussion regarding the "Repetition of HIIT workouts" (Line 372) as a barrier could be deepened. You mention that students found it monotonous, yet retention was high. It would be valuable to discuss this paradox further. Does the social element or the peer-coaching aspect mitigate the boredom of repetitive exercises? Additionally, on line 424, you suggest the intensity might be "slightly lower than expected." I would argue that for a school-based, peer-led intervention, a mean HR of 71% HRmax is actually quite successful. You might frame this more positively as a demonstration of feasibility in a non-laboratory setting, rather than a failure to meet strict laboratory HIIT criteria which are often unrealistic in schools.

Finally, regarding the "Unintended Consequences" (Line 196 and 363), you list the school-wide sports event disrupting dosage as an unintended consequence. In the context of process evaluation, this is perhaps better categorized strictly as a contextual barrier or implementation failure rather than an "unintended consequence" of the intervention itself (which usually refers to adverse events or unexpected positive side effects). Reconsidering this categorization might align better with the MRC framework's intent. Addressing these points will clarify the contribution of this work to the field of implementation science in youth physical activity.

**********

6. PLOS authors have the option to publish the peer review history of their article (what does this mean?). If published, this will include your full peer review and any attached files.

If you choose “no”, your identity will remain anonymous but your review may still be made public.

**Do you want your identity to be public for this peer review?**  For information about this choice, including consent withdrawal, please see our Privacy Policy.

Reviewer #1: No

Reviewer #2: **Yes:**  Alston Choong

**********

[NOTE: If reviewer comments were submitted as an attachment file, they will be attached to this email and accessible via the submission site. Please log into your account, locate the manuscript record, and check for the action link "View Attachments". If this link does not appear, there are no attachment files.]

To ensure your figures meet our technical requirements, please review our figure guidelines: https://journals.plos.org/plosone/s/figures

You may also use PLOS’s free figure tool, NAAS, to help you prepare publication quality figures: https://journals.plos.org/plosone/s/figures#loc-tools-for-figure-preparation.

NAAS will assess whether your figures meet our technical requirements by comparing each figure against our figure specifications.

# Author comment: Author response to Decision Letter 1

**DOI:** 10.1371/journal.pone.0352772.r002

11 Mar 2026

Dear reviewers and editor,

Thank you very much for your efforts in reviewing and processing our manuscript.

We have now addressed your comments and concerns and sent it back to you for your re-consideration.

For reviewers' feedback, we have uploaded a response to reviewers as required.

We have used PACE to manage our figures and uploaded in the system.

We have added the captions for the supporting files by the end of our manuscript.

We confirm that we have attained participants and teachers consent for taking part in the focus groups.

We hope our response have addressed your concerns. However, please do provide additional comments to help us develop a better version.

Best wishes,

Yong

Attachment Submitted filename: Response to reviewers.docx

# Peer review: Decision Letter 1

**DOI:** 10.1371/journal.pone.0352772.r003

**Authors:** Esedullah Akaras, Esedullah Akaras

6 Apr 2026

PONE-D-25-62980R1

Understanding the “how” and “why”: a mixed methods process evaluation for the PRO-HIIT intervention

PLOS One

Dear Dr. Williams,

Thank you for submitting your manuscript to PLOS ONE. After careful consideration, we feel that it has merit but does not fully meet PLOS ONE’s publication criteria as it currently stands. Therefore, we invite you to submit a revised version of the manuscript that addresses the points raised during the review process.

Please submit your revised manuscript by May 21 2026 11:59PM. If you will need more time than this to complete your revisions, please reply to this message or contact the journal office at plosone@plos.org. When you're ready to submit your revision, log on to https://www.editorialmanager.com/pone/ and select the 'Submissions Needing Revision' folder to locate your manuscript file.

Please include the following items when submitting your revised manuscript:

- A letter that responds to each point raised by the academic editor and reviewer(s). You should upload this letter as a separate file labeled 'Response to Reviewers'.
- A marked-up copy of your manuscript that highlights changes made to the original version. You should upload this as a separate file labeled 'Revised Manuscript with Track Changes'.
- An unmarked version of your revised paper without tracked changes. You should upload this as a separate file labeled 'Manuscript'.

If you would like to make changes to your financial disclosure, please include your updated statement in your cover letter. Guidelines for resubmitting your figure files are available below the reviewer comments at the end of this letter.

If applicable, we recommend that you deposit your laboratory protocols in protocols.io to enhance the reproducibility of your results. Protocols.io assigns your protocol its own identifier (DOI) so that it can be cited independently in the future. For instructions see: https://journals.plos.org/plosone/s/submission-guidelines#loc-laboratory-protocols. Additionally, PLOS ONE offers an option for publishing peer-reviewed Lab Protocol articles, which describe protocols hosted on protocols.io. Read more information on sharing protocols at https://plos.org/protocols?utm_medium=editorial-email&utm_source=authorletters&utm_campaign=protocols.

As the corresponding author, your ORCID iD is verified in the submission system and will appear in the published article. PLOS supports the use of ORCID, and we encourage all coauthors to register for an ORCID iD and use it as well. Please encourage your coauthors to verify their ORCID iD within the submission system before final acceptance, as unverified ORCID iDs will not appear in the published article. *Only*  the individual author can complete the verification step; PLOS staff *cannot*  verify ORCID iDs on behalf of authors.

We look forward to receiving your revised manuscript.

Kind regards,

Esedullah Akaras

Academic Editor

PLOS One

Journal Requirements:

1. If the reviewer comments include a recommendation to cite specific previously published works, please review and evaluate these publications to determine whether they are relevant and should be cited. There is no requirement to cite these works unless the editor has indicated otherwise.

2. Please review your reference list to ensure that it is complete and correct. If you have cited papers that have been retracted, please include the rationale for doing so in the manuscript text, or remove these references and replace them with relevant current references. Any changes to the reference list should be mentioned in the rebuttal letter that accompanies your revised manuscript. If you need to cite a retracted article, indicate the article’s retracted status in the References list and also include a citation and full reference for the retraction notice.

[Note: HTML markup is below. Please do not edit.]

Reviewers' comments:

Reviewer's Responses to Questions

**Comments to the Author**

1. If the authors have adequately addressed your comments raised in a previous round of review and you feel that this manuscript is now acceptable for publication, you may indicate that here to bypass the “Comments to the Author” section, enter your conflict of interest statement in the “Confidential to Editor” section, and submit your "Accept" recommendation.

Reviewer #1: (No Response)

Reviewer #2: (No Response)

**********

2. Is the manuscript technically sound, and do the data support the conclusions?

The manuscript must describe a technically sound piece of scientific research with data that supports the conclusions. Experiments must have been conducted rigorously, with appropriate controls, replication, and sample sizes. The conclusions must be drawn appropriately based on the data presented.

Reviewer #1: Yes

Reviewer #2: Yes

**********

3. Has the statistical analysis been performed appropriately and rigorously?

Reviewer #1: Yes

Reviewer #2: Yes

**********

4. Have the authors made all data underlying the findings in their manuscript fully available?

The PLOS Data policy requires authors to make all data underlying the findings described in their manuscript fully available without restriction, with rare exception (please refer to the Data Availability Statement in the manuscript PDF file). The data should be provided as part of the manuscript or its supporting information, or deposited to a public repository. For example, in addition to summary statistics, the data points behind means, medians and variance measures should be available. If there are restrictions on publicly sharing data—e.g. participant privacy or use of data from a third party—those must be specified.

Reviewer #1: Yes

Reviewer #2: Yes

**********

5. Is the manuscript presented in an intelligible fashion and written in standard English?

PLOS ONE does not copyedit accepted manuscripts, so the language in submitted articles must be clear, correct, and unambiguous. Any typographical or grammatical errors should be corrected at revision, so please note any specific errors here.

Reviewer #1: Yes

Reviewer #2: Yes

**********

6. Review Comments to the Author

Please use the space provided to explain your answers to the questions above. You may also include additional comments for the author, including concerns about dual publication, research ethics, or publication ethics. (Please upload your review as an attachment if it exceeds 20,000 characters)

Reviewer #1: Thank you very much for your response to my comments and for making certain amendments to the new version of the manuscript with the aim of accepting my suggestions. I understand that some of my suggestions were hard to accept, considering the fact that you already have extensive list of publications in which this type of training has been designed and investigated. However, I do not agree with some of your comments made. Anyway, I recommend acceptance of the manuscript as you probably would not be willing to change your mind in accordance with my proposals.

1. The control of intensity during HIIT (but real HIIT) in school-based settings is not unfeasible. I do it all the time and it works perfectly if running is the exercise modality.

2. The fact that “body-weight, multi-joint, cyclic activities are commonly adopted in school interventions and have been widely used in previous school-based HIIT research” does not mean that they are better or that they are optimal.

3. The criterium of time spent ≥90% VO2max is widely accepted criterium and it is recognized as a key physiological stimulus with physiological rationale backing it up (Odden et al., 2024; Molmen & Ronnestand, 2024). There are other references as well, of course. Other criteria are arbitrarily defined and that is why they are inferior to the one mentioned above.

Odden, I., Nymoen, L., Urianstad, T., Kristoffersen, M., Hammarström, D., Hansen, J., Mølmen, K.S., & Rønnestad, B.R. (2024). The higher the fraction of maximal oxygen uptake is during interval training, the greater is the cycling performance gain. European Journal of Sport Science, 24(11), 1583-1596.

Mølmen, K.S. & Rønnestad, B.R. (2024). A narrative review exploring advances in interval training for endurance athletes. Applied Physiology, Nutrition, and Metabolism, 49(7), 1008-1013.

4. Even though your primary aim was not to assess physiological adaptations, the training program investigated needs to be properly prescribed. If the program is not prescribed properly, then everything else is false. All results presented in your study are basically not related to HIIT, but to some other type of training.

5. Claiming that this type of HIIT (which is not HIIT) is more feasible, or only feasible, in school-setting is wrong. Real HIIT sessions, which are optimal for improvement of VO2max which is the main health-related physical fitness component, are very easily incorporated withing school settings. Studies which claim that inferior types of HIIT are more feasible and optimal are creating “noise” in the literature and offer inferior practical solutions. HIIT is HIIT. There is no school-based HIIT literature. Or there shouldn’t be school-based HIIT literature.

6. The fact that you used 220-age to assess HRmax is unacceptable for scientific publication. Check the variability in such estimation, please. I understand that estimation is more feasible, but some things need to be done properly.

7. You claim that “It is also not feasible to retain children and adolescent participants under such high exercise intensity”. It is feasible, you just need to prescribe exercise better to make it happen. That is the main purpose of being a coach or a PE teacher. And to add to that, intensity has been recognized as the key variable for health enhancement (Schwendinger et al., 2025), so trying to make students work at higher intensity, but with keeping the RPE low, is the ultimate goal in finding the right HIIT design.

Schwendinger, F., Infanger, D., Lichtenstein, E., Hinrichs, T., Knaier, R., Rowlands, A. V., & Schmidt-Trucksäss, A. (2025). Intensity or volume: the role of physical activity in longevity. European Journal of Preventive Cardiology, 32(1), 10–19.

8. The fact that Buchheit and Laursen’s paper is over a decade old does not make it obsolete. It does not change the fact that the propositions presented do not still stand. Again, saying that there is heterogeneity in HIIT studies is making “noise” and creating confusion. However, the main issue is that no optimal practical solutions are provided when poor program designs are labeled as HIIT.

9. The ≥90% VO2max/HRmax should be accepted as the main criterium and, as I pointed out earlier, we need to find the exercise design in which time ≥90% HRmax would be maximized but lowest levels of RPE and blood lactate and highest level of enjoyment and affect. Choosing the right exercises is the first step. And the choice should not be left to students to decide.

Reviewer #2: Dear Authors,

Thank you for your revised manuscript that shows substantive efforts made to address the methodological and interpretative concerns raised during the previous peer review process. The clarification of the high-intensity interval training definition within a school-based context, as opposed to a strictly controlled laboratory environment, significantly elevates the practical relevance of the work. Furthermore, the improved reporting of the heart rate intensity thresholds and the recategorization of the school sports event as a contextual barrier rather than an unintended consequence enhance the precision and transparency of the process evaluation narrative. The expanded limitations section appropriately contextualizes the small qualitative sample size, ensuring the findings are interpreted with suitable academic caution.

Addressing the methodological descriptions of the intervention delivery, the decision to omit the SAAFE principles from the peer coach training is adequately justified in the rebuttal. However, this creates a minor gap in the fidelity assessment narrative that warrants brief discussion. If peer coaches are responsible for demonstrating and leading the exercises but are not formally trained in the supportive or autonomous principles, this lack of training directly influences the "unknown" or "low" compliance scores for those specific SAAFE domains observed in Table 1. Adding a brief sentence in the discussion section that explicitly connects the limited scope of the peer coach training to these specific fidelity outcomes would provide a more cohesive and comprehensive interpretation of the implementation barriers.

Finally, while the main text is written in clear and standard English, a meticulous review of the bibliography reveals several formatting inconsistencies and metadata extraction errors indicative of unverified reference management software output. Submitting a manuscript at this advanced stage without a thorough manual proofread of the references does not align with the rigorous publication standards expected by the journal. For instance, Reference 5 displays the journal name in all capital letters and lacks standard page numbers. Reference 37 contains a formatting anomaly in the author name, listing "K BO" instead of the correct format. Reference 35 utilizes inconsistent capitalization for the journal title, and Reference 41 is missing volume and page numbers entirely. A manual, line-by-line proofread of the entire reference list is required to ensure all citations strictly adhere to the formatting guidelines, presenting a polished and professional final manuscript. Implementing these minor adjustments will ensure the manuscript perfectly reflects the high quality of the underlying research.

**********

7. PLOS authors have the option to publish the peer review history of their article (what does this mean?). If published, this will include your full peer review and any attached files.

If you choose “no”, your identity will remain anonymous but your review may still be made public.

**Do you want your identity to be public for this peer review?**  For information about this choice, including consent withdrawal, please see our Privacy Policy.

Reviewer #1: No

Reviewer #2: **Yes:**  Alston Choong

**********

[NOTE: If reviewer comments were submitted as an attachment file, they will be attached to this email and accessible via the submission site. Please log into your account, locate the manuscript record, and check for the action link "View Attachments". If this link does not appear, there are no attachment files.]

To ensure your figures meet our technical requirements, please review our figure guidelines: https://journals.plos.org/plosone/s/figures

You may also use PLOS’s free figure tool, NAAS, to help you prepare publication quality figures: https://journals.plos.org/plosone/s/figures#loc-tools-for-figure-preparation.

NAAS will assess whether your figures meet our technical requirements by comparing each figure against our figure specifications.

# Author comment: Author response to Decision Letter 2

**DOI:** 10.1371/journal.pone.0352772.r004

19 May 2026

19 May 2026

Dear reviewers,

RE: REVISION, PLOS ONE, PONE-D-25-62980 “Understanding the “how” and “why”: a mixed methods process evaluation for the PRO-HIIT intervention”.

Thank you very much again for your thorough review of our article. Similar to the revision one, we repeated your comments and provided our response accordingly. All revisions made to the manuscript have been highlighted in yellow, and the relevant revised text is repeated in this document in italics, where appropriate.

We look forward to hearing your thoughts on our revised manuscript.

Kind regards,

The authors

Reviewer #1:

Thank you very much for your response to my comments and for making certain amendments to the new version of the manuscript with the aim of accepting my suggestions. I understand that some of my suggestions were hard to accept, considering the fact that you already have extensive list of publications in which this type of training has been designed and investigated. However, I do not agree with some of your comments made. Anyway, I recommend acceptance of the manuscript as you probably would not be willing to change your mind in accordance with my proposals.

1. The control of intensity during HIIT (but real HIIT) in school-based settings is not unfeasible. I do it all the time and it works perfectly if running is the exercise modality.

2. The fact that “body-weight, multi-joint, cyclic activities are commonly adopted in school interventions and have been widely used in previous school-based HIIT research” does not mean that they are better or that they are optimal.

3. The criterium of time spent ≥90% VO2max is widely accepted criterium and it is recognized as a key physiological stimulus with physiological rationale backing it up (Odden et al., 2024; Molmen & Ronnestand, 2024). There are other references as well, of course. Other criteria are arbitrarily defined and that is why they are inferior to the one mentioned above.

Odden, I., Nymoen, L., Urianstad, T., Kristoffersen, M., Hammarström, D., Hansen, J., Mølmen, K.S., & Rønnestad, B.R. (2024). The higher the fraction of maximal oxygen uptake is during interval training, the greater is the cycling performance gain. European Journal of Sport Science, 24(11), 1583-1596.

Mølmen, K.S. & Rønnestad, B.R. (2024). A narrative review exploring advances in interval training for endurance athletes. Applied Physiology, Nutrition, and Metabolism, 49(7), 1008-1013.

4. Even though your primary aim was not to assess physiological adaptations, the training program investigated needs to be properly prescribed. If the program is not prescribed properly, then everything else is false. All results presented in your study are basically not related to HIIT, but to some other type of training.

5. Claiming that this type of HIIT (which is not HIIT) is more feasible, or only feasible, in school-setting is wrong. Real HIIT sessions, which are optimal for improvement of VO2max which is the main health-related physical fitness component, are very easily incorporated withing school settings. Studies which claim that inferior types of HIIT are more feasible and optimal are creating “noise” in the literature and offer inferior practical solutions. HIIT is HIIT. There is no school-based HIIT literature. Or there shouldn’t be school-based HIIT literature.

6. The fact that you used 220-age to assess HRmax is unacceptable for scientific publication. Check the variability in such estimation, please. I understand that estimation is more feasible, but some things need to be done properly.

7. You claim that “It is also not feasible to retain children and adolescent participants under such high exercise intensity”. It is feasible, you just need to prescribe exercise better to make it happen. That is the main purpose of being a coach or a PE teacher. And to add to that, intensity has been recognized as the key variable for health enhancement (Schwendinger et al., 2025), so trying to make students work at higher intensity, but with keeping the RPE low, is the ultimate goal in finding the right HIIT design.

Schwendinger, F., Infanger, D., Lichtenstein, E., Hinrichs, T., Knaier, R., Rowlands, A. V., & Schmidt-Trucksäss, A. (2025). Intensity or volume: the role of physical activity in longevity. European Journal of Preventive Cardiology, 32(1), 10–19.

8. The fact that Buchheit and Laursen’s paper is over a decade old does not make it obsolete. It does not change the fact that the propositions presented do not still stand. Again, saying that there is heterogeneity in HIIT studies is making “noise” and creating confusion. However, the main issue is that no optimal practical solutions are provided when poor program designs are labeled as HIIT.

9. The ≥90% VO2max/HRmax should be accepted as the main criterium and, as I pointed out earlier, we need to find the exercise design in which time ≥90% HRmax would be maximized but lowest levels of RPE and blood lactate and highest level of enjoyment and affect. Choosing the right exercises is the first step. And the choice should not be left to students to decide.

Response:

We greatly appreciate your acceptance of our manuscript despite our differences on several points. To our knowledge, no publication has claimed or argued that only running and cycling should be classified as HIIT, or that an intensity of ≤90% VO₂max is insufficient for classification as HIIT. In addition, there is not one single marker of intensity, with HR, RPE, and affect all widely used in the literature. Likewise, the presence of school-based HIIT literature has not been disputed. As you may be also aware, numerous HIIT reviews have reported that resistance- and game-based HIIT are commonly adopted exercise modalities and are becoming increasingly popular as they offer further flexibility and adaptability to school-based programmes, compared to traditional running- or cycling-based HIIT. This evolution has contributed to the implementation and scalability of school-based interventions and are important from a public health perspective.

Indeed, the presence of these exercise modalities does not imply that they are superior or optimal, they just provide alternative options. Therefore, unless and until scientific evidence demonstrates, this area of research, including the current study, should not be described as “noise,” but rather as part of the continued development of contemporary HIIT literature.

Despite these differing perspectives we look forward to future scientific debate on this topic.

Regarding your comments on the estimation of HRmax, we fully acknowledge the limitations of using the “220-age” equation. However, as stated in the manuscript, resource limitations prevented us from objectively measuring HRmax for each participant. We also recognize that there are other alternative estimation formulas that may provide improved accuracy, such as 208–0.7*age. Nevertheless, because we used Polar devices, which apply the 220-age equation to determine HR zones (e.g., Zone 4 represents 81–90% HRmax; see https://www.polar.com/en/guide/calculate-max-heart rate?srsltid=AfmBOorCHY7B_AJAbmBk_5CQ0vm27Fjy-LrCXYtRi5bmoryobQvQ6nsq), we considered it appropriate to maintain consistency with this calculation method.

Reviewer #2:

Reviewer #2: Dear Authors,

Thank you for your revised manuscript that shows substantive efforts made to address the methodological and interpretative concerns raised during the previous peer review process. The clarification of the high-intensity interval training definition within a school-based context, as opposed to a strictly controlled laboratory environment, significantly elevates the practical relevance of the work. Furthermore, the improved reporting of the heart rate intensity thresholds and the recategorization of the school sports event as a contextual barrier rather than an unintended consequence enhance the precision and transparency of the process evaluation narrative. The expanded limitations section appropriately contextualizes the small qualitative sample size, ensuring the findings are interpreted with suitable academic caution.

Response:

Thank you very much for your positive words; your insightful suggestions have improved the manuscript.

• Addressing the methodological descriptions of the intervention delivery, the decision to omit the SAAFE principles from the peer coach training is adequately justified in the rebuttal. However, this creates a minor gap in the fidelity assessment narrative that warrants brief discussion. If peer coaches are responsible for demonstrating and leading the exercises but are not formally trained in the supportive or autonomous principles, this lack of training directly influences the "unknown" or "low" compliance scores for those specific SAAFE domains observed in Table 1. Adding a brief sentence in the discussion section that explicitly connects the limited scope of the peer coach training to these specific fidelity outcomes would provide a more cohesive and comprehensive interpretation of the implementation barriers.

Response:

Thank you for the constructive suggestion. Indeed, we only provided training of SAAFE principles among PE teachers and research assistants who conducted the data collection. We have added this in the discussion in lines 464 to 469, as shown below:

It should be noted that the peer coaches did not receive training in the SAAFE principles during the training workshop, and we did not conduct on-site observations to evaluate the implementation of the principles in practice among PE teachers or researcher assistants (e.g., encourage mutual support or minimise controlling language). Therefore, the compliance to some of these principles remains unknown or low, which should not be overlooked in future investigations.

Finally, while the main text is written in clear and standard English, a meticulous review of the bibliography reveals several formatting inconsistencies and metadata extraction errors indicative of unverified reference management software output. Submitting a manuscript at this advanced stage without a thorough manual proofread of the references does not align with the rigorous publication standards expected by the journal. For instance, Reference 5 displays the journal name in all capital letters and lacks standard page numbers. Reference 37 contains a formatting anomaly in the author name, listing "K BO" instead of the correct format. Reference 35 utilizes inconsistent capitalization for the journal title, and Reference 41 is missing volume and page numbers entirely. A manual, line-by-line proofread of the entire reference list is required to ensure all citations strictly adhere to the formatting guidelines, presenting a polished and professional final manuscript. Implementing these minor adjustments will ensure the manuscript perfectly reflects the high quality of the underlying research.

Response:

We greatly appreciate your careful review of the reference list. We have reviewed and made changes accordingly, as listed below.

5. Hay J, Maximova K, Durksen A, Carson V, Rinaldi RL, Torrance B, et al. Physical activity intensity and cardiometabolic risk in youth. Archives of Pediatrics & Adolescent Medicine. 2012;166(11):1022–1029.

15. Lubans DR, Lonsdale C, Cohen K, Eather N, Beauchamp MR, Morgan PJ, et al. Framework for the design and delivery of organized physical activity sessions for children and adolescents: rationale and description of the ‘SAAFE’ teaching principles. International Journal of Behavioral Nutrition and Physical Activity. 2017;14(1):1-11.

19. Kennedy SG, Leahy AA, Smith JJ, Eather N, Hillman CH, Morgan PJ, et al. Process Evaluation of a School-Based High-Intensity Interval Training Program for Older Adolescents: The Burn 2 Learn Cluster Randomised Controlled Trial. Children (Basel). 2020;7(12):299.

20. Liu Y, Williams CA, Weston KL, Duncombe SL, Malik AA, Barker AR. Validation and calibration for embedding rating of perceived exertion into high-intensity interval exercise in adolescents: a lab-based study. Pediatric Exercise Science. 2024;37(1):11-17.

21. Liu Y, Barker AR, Li M, Gu Y, Williams CA. Validation of polar verity sense for heart rate monitoring during school-based high-intensity interval training in adolescents. Journal of Sports Sciences. 2025;43(11):1076-1084.

29. Lubans DR, Smith JJ, Eather N, Leahy AA, Morgan PJ, Lonsdale C, et al. Time-efficient intervention to improve older adolescents' cardiorespiratory fitness: findings from the 'Burn 2 Learn' cluster randomised controlled trial. British Journal of Sports Medicine. 2021;55(13):751–758.

33. Weston KL, Innerd A, Azevedo LB, Bock S, Batterham AM. Process Evaluation of Project FFAB (Fun Fast Activity Blasts): A Multi-Activity School-Based High-Intensity Interval Training Intervention. Frontiers in Sports and Active Living. 2021;3:737900.

36. Owen KB, Smith J, Lubans DR, Ng JY, Lonsdale C. Self-determined motivation and physical activity in children and adolescents: a systematic review and meta-analysis. Preventive Medicine. 2014;67:270-9.

40. Li M, Liu Y, Gu Y, Sum RKW. Physical activity interventions among children and adolescents in China: a scoping review through an equity lens. International Journal of Behavioral Nutrition and Physical Activity. 2025;23(1):9.

Attachment Submitted filename: Responses_Reviewer2_PONE-D-25-62980R1.docx

# Peer review: Decision Letter 2

**DOI:** 10.1371/journal.pone.0352772.r005

**Authors:** Esedullah Akaras, Esedullah Akaras, Esedullah Akaras

4 Jun 2026

PONE-D-25-62980R2

Understanding the “how” and “why”: a mixed methods process evaluation for the PRO-HIIT intervention

PLOS One

Dear Dr. Williams,

Thank you for submitting your manuscript to PLOS ONE. After careful consideration, we feel that it has merit but does not fully meet PLOS ONE’s publication criteria as it currently stands. Therefore, we invite you to submit a revised version of the manuscript that addresses the points raised during the review process.

Please submit your revised manuscript by Jul 19 2026 11:59PM. If you will need more time than this to complete your revisions, please reply to this message or contact the journal office at plosone@plos.org. When you're ready to submit your revision, log on to https://www.editorialmanager.com/pone/ and select the 'Submissions Needing Revision' folder to locate your manuscript file.

Please include the following items when submitting your revised manuscript:

- A letter that responds to each point raised by the academic editor and reviewer(s). You should upload this letter as a separate file labeled 'Response to Reviewers'.
- A marked-up copy of your manuscript that highlights changes made to the original version. You should upload this as a separate file labeled 'Revised Manuscript with Track Changes'.
- An unmarked version of your revised paper without tracked changes. You should upload this as a separate file labeled 'Manuscript'.

If you would like to make changes to your financial disclosure, please include your updated statement in your cover letter. Guidelines for resubmitting your figure files are available below the reviewer comments at the end of this letter.

If applicable, we recommend that you deposit your laboratory protocols in protocols.io to enhance the reproducibility of your results. Protocols.io assigns your protocol its own identifier (DOI) so that it can be cited independently in the future. For instructions see: https://journals.plos.org/plosone/s/submission-guidelines#loc-laboratory-protocols. Additionally, PLOS ONE offers an option for publishing peer-reviewed Lab Protocol articles, which describe protocols hosted on protocols.io. Read more information on sharing protocols at https://plos.org/protocols?utm_medium=editorial-email&utm_source=authorletters&utm_campaign=protocols.

As the corresponding author, your ORCID iD is verified in the submission system and will appear in the published article. PLOS supports the use of ORCID, and we encourage all coauthors to register for an ORCID iD and use it as well. Please encourage your coauthors to verify their ORCID iD within the submission system before final acceptance, as unverified ORCID iDs will not appear in the published article. *Only*  the individual author can complete the verification step; PLOS staff *cannot*  verify ORCID iDs on behalf of authors.

We look forward to receiving your revised manuscript.

Kind regards,

Esedullah Akaras

Academic Editor

PLOS One

Journal Requirements:

1. If the reviewer comments include a recommendation to cite specific previously published works, please review and evaluate these publications to determine whether they are relevant and should be cited. There is no requirement to cite these works unless the editor has indicated otherwise.

2. Please review your reference list to ensure that it is complete and correct. If you have cited papers that have been retracted, please include the rationale for doing so in the manuscript text, or remove these references and replace them with relevant current references. Any changes to the reference list should be mentioned in the rebuttal letter that accompanies your revised manuscript. If you need to cite a retracted article, indicate the article’s retracted status in the References list and also include a citation and full reference for the retraction notice.

[Note: HTML markup is below. Please do not edit.]

Reviewer's Responses to Questions

**Comments to the Author**

1. If the authors have adequately addressed your comments raised in a previous round of review and you feel that this manuscript is now acceptable for publication, you may indicate that here to bypass the “Comments to the Author” section, enter your conflict of interest statement in the “Confidential to Editor” section, and submit your "Accept" recommendation.

Reviewer #2: (No Response)

**********

2. Is the manuscript technically sound, and do the data support the conclusions?

The manuscript must describe a technically sound piece of scientific research with data that supports the conclusions. Experiments must have been conducted rigorously, with appropriate controls, replication, and sample sizes. The conclusions must be drawn appropriately based on the data presented.

Reviewer #2: Yes

**********

3. Has the statistical analysis been performed appropriately and rigorously?

Reviewer #2: Yes

**********

4. Have the authors made all data underlying the findings in their manuscript fully available?

The PLOS Data policy requires authors to make all data underlying the findings described in their manuscript fully available without restriction, with rare exception (please refer to the Data Availability Statement in the manuscript PDF file). The data should be provided as part of the manuscript or its supporting information, or deposited to a public repository. For example, in addition to summary statistics, the data points behind means, medians and variance measures should be available. If there are restrictions on publicly sharing data—e.g. participant privacy or use of data from a third party—those must be specified.

Reviewer #2: Yes

**********

5. Is the manuscript presented in an intelligible fashion and written in standard English?

PLOS ONE does not copyedit accepted manuscripts, so the language in submitted articles must be clear, correct, and unambiguous. Any typographical or grammatical errors should be corrected at revision, so please note any specific errors here.

Reviewer #2: Yes

**********

6. Review Comments to the Author

Please use the space provided to explain your answers to the questions above. You may also include additional comments for the author, including concerns about dual publication, research ethics, or publication ethics. (Please upload your review as an attachment if it exceeds 20,000 characters)

Reviewer #2: Dear Authors,

Thank you for submitting the revised manuscript that has presented a highly valuable, pragmatic, and rigorously evaluated school-based health promotion study. Conducting a process evaluation using the Medical Research Council (MRC) guidance is an excellent methodological choice that provides critical context to the trial outcomes. The authors provide a robust, evidence-based rebuttal to the previous reviewer regarding the definition of High-Intensity Interval Training (HIIT) in a school setting, which is highly commendable. From the perspective of sports and exercise medicine, the assertion that pedagogical feasibility, student enjoyment, and pragmatic adaptations (such as bodyweight resistance exercises and age-based heart rate estimations) are vital for the real-world translation and scalability of public health interventions is fully supported. The authors' defense was scientifically sound and professionally articulated. The manuscript is in excellent shape; however, to ensure full compliance with the highest international reporting standards, a few minor methodological refinements have been identified that will further elevate the quality of this work.

In the Introduction, the research gap has been effectively and clearly defined. Process evaluations are indeed frequently undervalued in sports science, and the rationale for adopting the MRC framework is compelling. The transition from the overarching concept of school-based HIIT to the specific need for evaluating the PRO-HIIT implementation is logical and well-justified.

In the Methods section, the detailed mapping of the process evaluation measures to the MRC framework is a significant strength. However, a minor methodological flaw remains regarding the explicit classification of the mixed methods approach. While it is correctly stated that both quantitative and qualitative data were collected, the manuscript lacks a formal declaration of the specific mixed methods design typology employed (for instance, a convergent parallel design, an explanatory sequential design, or an exploratory sequential design). To align with the Good Reporting of A Mixed Methods Study (GRAMMS) framework, it is recommended that the authors explicitly define their mixed methods design in the Data Analysis section. A brief explanation of how and at what stage the quantitative and qualitative data were integrated to formulate the conclusions should be provided.

Furthermore, while the qualitative methodology involving focus groups and thematic analysis (using the Braun and Clarke approach) is sound, there is no explicit mention of adherence to the Consolidated Criteria for Reporting Qualitative Research (COREQ), which is the standard EQUATOR Network guideline for qualitative studies. As an actionable recommendation, the authors should insert a brief statement confirming adherence to the COREQ guidelines. In doing so, the concept of data saturation within the qualitative data subsection must be briefly addressed. Given the small qualitative sample size (nine students and two teachers), it is important to explicitly state whether thematic saturation was reached or if the findings represent an exploratory thematic overview rather than an exhaustively saturated dataset. This transparency will protect the manuscript from future methodological critiques regarding qualitative sample size adequacy.

The Results and Discussion sections are meticulously constructed. The integration of the quantitative dose-delivered metrics with the qualitative contextual barriers provides a rich, nuanced picture of school-based intervention realities. The incorporation of the previous reviewer's suggestion regarding the lack of Supportive, Active, Autonomous, Fair, and Enjoyable (SAAFE) principle training for the peer coaches is greatly appreciated. This addition provides a highly transparent and cohesive explanation for the fidelity outcomes observed in Table 1. The discussion appropriately balances the physiological ideals of HIIT with the sociological and pedagogical realities of the school environment.

In conclusion, this is a highly commendable manuscript that makes a significant contribution to the literature on physical activity interventions in adolescents. The recommendation for a minor revision is strictly to allow the authors to explicitly align their methodology section with the GRAMMS and COREQ reporting guidelines, specifically by naming their mixed methods design typology and clarifying qualitative thematic saturation, thereby ensuring full compliance with the EQUATOR Network's international reporting standards.

**********

7. PLOS authors have the option to publish the peer review history of their article (what does this mean?). If published, this will include your full peer review and any attached files.

If you choose “no”, your identity will remain anonymous but your review may still be made public.

**Do you want your identity to be public for this peer review?**  For information about this choice, including consent withdrawal, please see our Privacy Policy.

Reviewer #2: **Yes:**  Alston Choong

**********

[NOTE: If reviewer comments were submitted as an attachment file, they will be attached to this email and accessible via the submission site. Please log into your account, locate the manuscript record, and check for the action link "View Attachments". If this link does not appear, there are no attachment files.]

To ensure your figures meet our technical requirements, please review our figure guidelines: https://journals.plos.org/plosone/s/figures

You may also use PLOS’s free figure tool, NAAS, to help you prepare publication quality figures: https://journals.plos.org/plosone/s/figures#loc-tools-for-figure-preparation.

NAAS will assess whether your figures meet our technical requirements by comparing each figure against our figure specifications.

# Author comment: Author response to Decision Letter 3

**DOI:** 10.1371/journal.pone.0352772.r006

9 Jun 2026

9 June 2026

RE: REVISION, PLOS ONE, PONE-D-25-62980 “Understanding the “how” and “why”: a mixed methods process evaluation for the PRO-HIIT intervention”.

To the Editor and Dr. Alston Choong [named reviewer],

We sincerely thank you for your positive and thoughtful evaluation of our manuscript. We greatly appreciate the recognition of the value of the process evaluation, the application of the Medical Research Council (MRC) framework, and the rationale underpinning the design and implementation of the PRO-HIIT intervention.

We appreciate your constructive recommendations regarding alignment with the Good Reporting of A Mixed Methods Study (GRAMMS) framework and the Consolidated Criteria for Reporting Qualitative Research (COREQ) guidelines. In response, we have revised the manuscript to further strengthen methodological transparency and reporting quality.

We have repeated your comments below only for those revisions are required and provided a response accordingly with changes to the manuscript were highlighted in yellow.

We hope we have satisfied your queries satisfactorily and we look forward to a decision about the revised manuscript.

Kind regards,

The authors

1. In the Methods section, the detailed mapping of the process evaluation measures to the MRC framework is a significant strength. However, a minor methodological flaw remains regarding the explicit classification of the mixed methods approach. While it is correctly stated that both quantitative and qualitative data were collected, the manuscript lacks a formal declaration of the specific mixed methods design typology employed (for instance, a convergent parallel design, an explanatory sequential design, or an exploratory sequential design). To align with the Good Reporting of A Mixed Methods Study (GRAMMS) framework, it is recommended that the authors explicitly define their mixed methods design in the Data Analysis section. A brief explanation of how and at what stage the quantitative and qualitative data were integrated to formulate the conclusions should be provided.

Response:

Thank you for this helpful suggestion and for highlighting the importance of explicitly reporting the mixed methods design in accordance with the GRAMMS framework. In response, we have revised the Data Analysis section to specify that the study employed a convergent parallel mixed methods design, whereby quantitative and qualitative data were collected concurrently, analysed independently, and integrated during the interpretation stage. We have also added a description of the integration process, clarifying that findings from both datasets were triangulated across the Medical Research Council (MRC) process evaluation domains and jointly interpreted to identify areas of convergence, complementarity, and divergence. These integrated findings informed the overall conclusions regarding the implementation, acceptability, and contextual influences of the PRO-HIIT intervention.

The Data Analysis section of the manuscript has been changed to:

The current study employed a convergent parallel mixed methods design. Quantitative data were collected for reach, recruitment and retention, dose delivered, fidelity, dose received and response. Data were analysed using IBM SPSS Statistics for Windows (SPSS 28.0; IBM Corporation, Armonk, NY, USA) unless specified. Qualitative data were collected for adaptation, response, dose received, unintended consequence, barriers and facilitators, and contamination. The focus group recordings were transcribed verbatim in Chinese and were translated to English by the first author. A second author (MHL) checked the transcribed data for completeness and accuracy. Transcripts were analysed using thematic analysis following a six-phase guidance (i.e., adaptation, response, barriers, facilitators, and contamination). The coding was initially deductive based on the MRC guidance, followed by an inductive process directed by the content of the data (25). To maximise consistency and completeness, two authors (YL and SLD) independently conducted the thematic analysis using NVivo software (Version 15) and discussed their findings until consensus was reached. Integration of quantitative and qualitative findings occurred during the interpretation stage. Areas of convergence, complementarity, and divergence between the quantitative and qualitative findings were jointly interpreted to inform the overall conclusions of the process evaluation.

2. Furthermore, while the qualitative methodology involving focus groups and thematic analysis (using the Braun and Clarke approach) is sound, there is no explicit mention of adherence to the Consolidated Criteria for Reporting Qualitative Research (COREQ), which is the standard EQUATOR Network guideline for qualitative studies. As an actionable recommendation, the authors should insert a brief statement confirming adherence to the COREQ guidelines. In doing so, the concept of data saturation within the qualitative data subsection must be briefly addressed. Given the small qualitative sample size (nine students and two teachers), it is important to explicitly state whether thematic saturation was reached or if the findings represent an exploratory thematic overview rather than an exhaustively saturated dataset. This transparency will protect the manuscript from future methodological critiques regarding qualitative sample size adequacy.

Response:

We thank the reviewer for this valuable suggestion regarding qualitative reporting transparency. We agree that the COREQ checklist provides important guidance for reporting qualitative research. However, the qualitative component of the present study was embedded within a broader mixed methods process evaluation and was not prospectively designed or reported using the COREQ framework. Therefore, we believe it would not be appropriate to retrospectively claim adherence to COREQ when the study was not originally conducted and documented according to its reporting criteria.

Nevertheless, we appreciate the reviewer's concern regarding qualitative sample adequacy and thematic saturation. In response, we have revised the Strengths and Limitations section to explicitly acknowledge that the qualitative sample comprised only nine students and two teachers and that exhaustive thematic saturation was not the primary objective of this process evaluation. We now clarify that the qualitative findings should be interpreted as an exploratory thematic overview intended to complement the quantitative process evaluation and provide contextual insights into implementation experiences, barriers, facilitators, and participant responses.

The Strength and Limitations section has been changed to:

The current study utilised a convergent parallel mixed methods approach, guided by the MRC framework to give a complete picture of the process evaluation. The integration of quantitative and qualitative data enabled triangulation across process evaluation domains and reduced the likelihood of incomplete reporting or biased interpretation. However, several limitations should be acknowledged. First, the students and teachers were not blinded to the intervention groups and the session intensity were only measured in a sub-sample of participants and in PE lessons only. This could potentially contaminate or obscure our research findings. Second, several process evaluation outcomes were assessed using subjective measures (e.g., session quality scores), which may have been susceptible to social desirability bias. Third, the qualitative sample comprised only nine students across two focus groups and two PE teachers who participated in individual interviews. Although these data provided valuable insights into participants’ experiences and perceptions of the intervention, the sample size may have limited the breadth and diversity of perspectives captured. The qualitative component was designed to complement the quantitative process evaluation rather than to achieve exhaustive thematic saturation (44). Therefore, the qualitative findings should be interpreted as an exploratory thematic overview of key implementation experiences, barriers, facilitators, and perceived impacts within this context.

3. In conclusion, this is a highly commendable manuscript that makes a significant contribution to the literature on physical activity interventions in adolescents. The recommendation for a minor revision is strictly to allow the authors to explicitly align their methodology section with the GRAMMS and COREQ reporting guidelines, specifically by naming their mixed methods design typology and clarifying qualitative thematic saturation, thereby ensuring full compliance with the EQUATOR Network's international reporting standards.

Response:

Thank you for your positive appraisal of our manuscript and for recognising its contribution to the literature on school-based physical activity interventions. We appreciate the constructive recommendations regarding reporting transparency and methodological clarity. In response, we have revised the manuscript to explicitly identify the study as a convergent parallel mixed methods design. We have also added an extra sentence to acknowledge the exploratory nature of the qualitative findings and to highlight future research directions.

The Conclusion section has been changed to:

The present study provides valuable insights into the “how” and “why” the PRO-HIIT intervention works or not within a Chinese secondary school setting, utilising a convergent parallel mixed methods process evaluation. Overall, the PRO-HIIT intervention demonstrated favourable reach, retention, and dose received as indicated by the positive responses from both teachers and students. However, the dose delivered fell slightly below the predefined threshold mainly due to uncontrollable competing priorities, and implementation fidelity was only partially achieved as intended. Our study demonstrated the potential for incorporating HIIT in existing PA curriculum time to engage Chinese children and adolescents in vigorous PA. As a reflection, the implementation of the PRO-HIIT intervention may have come at the expense of students’ autonomy. Future studies should aim to better balance implementation fidelity and autonomy support and seek to foster PA motivation through intrinsic means. Given the exploratory nature of the qualitative component, further research involving larger and more diverse qualitative samples is warranted to confirm and extend the implementation insights identified in the present study.

Attachment Submitted filename: Response to Reviewers_v3.docx

# Peer review: Decision Letter 3

**DOI:** 10.1371/journal.pone.0352772.r007

**Authors:** Esedullah Akaras, Esedullah Akaras, Esedullah Akaras, Esedullah Akaras

15 Jun 2026

Understanding the “how” and “why”: a mixed methods process evaluation for the PRO-HIIT intervention

PONE-D-25-62980R3

Dear Dr. Williams,

We’re pleased to inform you that your manuscript has been judged scientifically suitable for publication and will be formally accepted for publication once it meets all outstanding technical requirements.

Within one week, you’ll receive an e-mail detailing the required amendments. When these have been addressed, you’ll receive a formal acceptance letter and your manuscript will be scheduled for publication.

An invoice will be generated when your article is formally accepted. Please note, if your institution has a publishing partnership with PLOS and your article meets the relevant criteria, all or part of your publication costs will be covered. Please make sure your user information is up-to-date by logging into Editorial Manager at Editorial Manager® and clicking the ‘Update My Information' link at the top of the page. For questions related to billing, please contact billing support.

If your institution or institutions have a press office, please notify them about your upcoming paper to help maximize its impact. If they’ll be preparing press materials, please inform our press team as soon as possible -- no later than 48 hours after receiving the formal acceptance. Your manuscript will remain under strict press embargo until 2 pm Eastern Time on the date of publication. For more information, please contact onepress@plos.org.

Kind regards,

Esedullah Akaras

Academic Editor

PLOS One

Additional Editor Comments (optional):

Reviewers' comments:

Reviewer's Responses to Questions

**Comments to the Author**

1. If the authors have adequately addressed your comments raised in a previous round of review and you feel that this manuscript is now acceptable for publication, you may indicate that here to bypass the “Comments to the Author” section, enter your conflict of interest statement in the “Confidential to Editor” section, and submit your "Accept" recommendation.

Reviewer #2: All comments have been addressed

**********

2. Is the manuscript technically sound, and do the data support the conclusions?

The manuscript must describe a technically sound piece of scientific research with data that supports the conclusions. Experiments must have been conducted rigorously, with appropriate controls, replication, and sample sizes. The conclusions must be drawn appropriately based on the data presented.

Reviewer #2: Yes

**********

3. Has the statistical analysis been performed appropriately and rigorously?

Reviewer #2: Yes

**********

4. Have the authors made all data underlying the findings in their manuscript fully available?

The PLOS Data policy requires authors to make all data underlying the findings described in their manuscript fully available without restriction, with rare exception (please refer to the Data Availability Statement in the manuscript PDF file). The data should be provided as part of the manuscript or its supporting information, or deposited to a public repository. For example, in addition to summary statistics, the data points behind means, medians and variance measures should be available. If there are restrictions on publicly sharing data—e.g. participant privacy or use of data from a third party—those must be specified.

Reviewer #2: Yes

**********

5. Is the manuscript presented in an intelligible fashion and written in standard English?

PLOS ONE does not copyedit accepted manuscripts, so the language in submitted articles must be clear, correct, and unambiguous. Any typographical or grammatical errors should be corrected at revision, so please note any specific errors here.

Reviewer #2: Yes

**********

6. Review Comments to the Author

Please use the space provided to explain your answers to the questions above. You may also include additional comments for the author, including concerns about dual publication, research ethics, or publication ethics. (Please upload your review as an attachment if it exceeds 20,000 characters)

Reviewer #2: Dear Authors,

Thank you for submitting the revised manuscript that investigates the process evaluation of a school-based high-intensity interval training intervention using a mixed methods approach. It is a pleasure to evaluate this revised manuscript. The authors are commended for their thorough, rigorous, and professional responses to the previous peer review concerns. The methodological and interpretative enhancements have significantly enhanced the manuscript's scientific integrity, bringing it into full alignment with the highest international standards for sports and performance science publications.

The reviewer notes that the explicit identification of the convergent parallel mixed-methods design within the Data Analysis section successfully meets the requirements of the Good Reporting of A Mixed Methods Study (GRAMMS) framework. Furthermore, the detailed explanation of how quantitative and qualitative findings were integrated and triangulated during the interpretation stage provides excellent methodological clarity. This addition firmly establishes the rigorous analytical framework utilized to derive the study's conclusions.

The authors are also commended for their transparent handling of the constraints in qualitative reporting. By explicitly acknowledging the small qualitative sample size in the Strengths and Limitations section and by clearly framing the thematic findings as an exploratory overview rather than claiming exhaustive thematic saturation, the manuscript now accurately reflects the scope and intent of the qualitative data. This careful contextualization effectively preempts methodological critiques regarding strict adherence to the Consolidated Criteria for Reporting Qualitative Research (COREQ) guidelines, ensuring that the qualitative insights are interpreted with the appropriate academic caution.

The manuscript is now methodologically robust and scientifically sound, and it addresses a highly relevant topic in public health and physical education. The pragmatic adaptations explored within this process evaluation provide highly valuable insights for the real-world translation and scalability of school-based interventions. No further revisions are required.

**********

7. PLOS authors have the option to publish the peer review history of their article (what does this mean?). If published, this will include your full peer review and any attached files.

If you choose “no”, your identity will remain anonymous but your review may still be made public.

**Do you want your identity to be public for this peer review?**  For information about this choice, including consent withdrawal, please see our Privacy Policy.

Reviewer #2: **Yes:**  Alston Choong

**********

# Editor report: Acceptance letter

**DOI:** 10.1371/journal.pone.0352772.r008

**Authors:** Esedullah Akaras, Esedullah Akaras, Esedullah Akaras, Esedullah Akaras

PONE-D-25-62980R3

PLOS One

Dear Dr. Williams,

I'm pleased to inform you that your manuscript has been deemed suitable for publication in PLOS One. Congratulations! Your manuscript is now being handed over to our production team.

At this stage, our production department will prepare your paper for publication. This includes ensuring the following:

* All references, tables, and figures are properly cited

* All relevant supporting information is included in the manuscript submission,

* There are no issues that prevent the paper from being properly typeset

You will receive further instructions from the production team, including instructions on how to review your proof when it is ready. Please keep in mind that we are working through a large volume of accepted articles, so please give us a few days to review your paper and let you know the next and final steps.

Lastly, if your institution or institutions have a press office, please let them know about your upcoming paper now to help maximize its impact. If they'll be preparing press materials, please inform our press team within the next 48 hours. Your manuscript will remain under strict press embargo until 2 pm Eastern Time on the date of publication. For more information, please contact onepress@plos.org.

You will receive an invoice from PLOS for your publication fee after your manuscript has reached the completed accept phase. If you receive an email requesting payment before acceptance or for any other service, this may be a phishing scheme. Learn how to identify phishing emails and protect your accounts at https://explore.plos.org/phishing.

If we can help with anything else, please email us at customercare@plos.org.

Thank you for submitting your work to PLOS ONE and supporting open access.

Kind regards,

PLOS ONE Editorial Office Staff

on behalf of

Dr. Esedullah Akaras

Academic Editor

PLOS One
